# Fluorinated and Non-Fluorinated 1,4-Diarylpyrazoles via MnO_2_-Mediated Mechanochemical Deacylative Oxidation of 5-Acylpyrazolines †

**DOI:** 10.3390/molecules27238446

**Published:** 2022-12-02

**Authors:** Greta Utecht-Jarzyńska, Anna Kowalczyk, Marcin Jasiński

**Affiliations:** 1 Department of Organic and Applied Chemistry, Faculty of Chemistry, University of Lodz, Tamka 12, 91403 Lodz, Poland; 2 Doctoral School of Exact and Natural Sciences, University of Lodz, Banacha 12/16, 90237 Lodz, Poland

**Keywords:** pyrazole, nitrile imine, mechanochemistry, (3 + 2)-cycloaddition, deacylation, oxidation

## Abstract

A solvent-free two-step synthesis of polyfunctionalized pyrazoles under ball-milling mechanochemical conditions was developed. The protocol comprises (3 + 2)-cycloaddition of in situ generated nitrile imines and chalcones, followed by oxidation of the initially formed 5-acylpyrazolines with activated MnO_2_. The second step proceeds via an exclusive deacylative pathway, to give a series of 1,4-diarylpyrazoles functionalized with a fluorinated (CF_3_) or non-fluorinated (Ph, COOEt, Ac) substituent at C(3) of the heterocyclic ring. In contrast, MnO_2_-mediated oxidation of a model isomeric 4-acylpyrazoline proceeded with low chemoselectivity, leading to fully substituted pyrazole as a major product formed via dehydrogenative aromatization. The presented approach extends the scope of the known methods carried out in organic solvents and enables the preparation of polyfunctionalized pyrazoles, which are of general interest in medicine and material sciences.

## 1. Introduction

**Figure 1:**
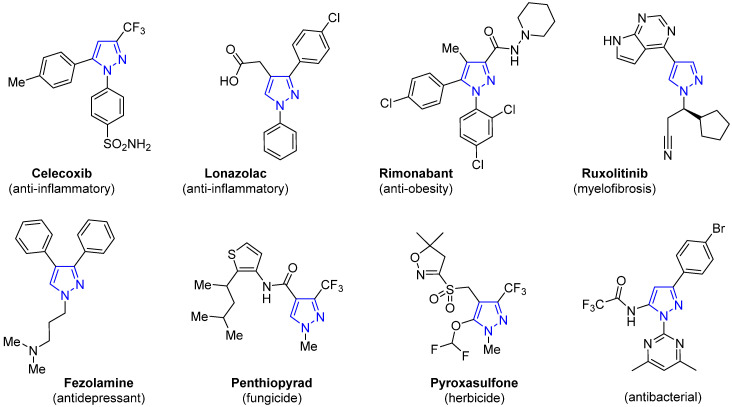
Structures of selected fluorinated and non-fluorinated pyrazole-based pharmaceuticals and agrochemicals.

Due to the discovery of a number of practical applications, there is increasing interest in the chemistry of pyrazole-based compounds, and fluorinated analogues are of special significance in medicine, crop protection, as well as material sciences [1,2,3,4]. The title heterocycle constitutes a key structural element of pharmaceuticals and agrochemicals; they exhibit a variety of biological activities such as being anti-inflammatory (e.g., Celecoxib, Lonazolac), antibacterial, anticancer (e.g., Crizotinib), anti-obesity (e.g., Rimonabant), antidepressant (e.g., Fezolamine), antiviral (e.g., Lenacapavir), and antifungal (e.g., Penthiopyrad), and have been widely applied as pesticides (Figure 1) [5,6,7,8,9,10,11,12,13]. In addition, some pyrazoles have been successfully applied in polymer chemistry, as well as for the preparation of advanced liquid crystalline materials [14,15]. Furthermore, polyfunctionalized pyrazoles can efficiently act as ligands in transition metal-catalyzed reactions [1,2,16]. Taking into account the general significance of this class of *N*-heterocycles, the development of new synthetic protocols to access pyrazoles with the desired substitution patterns is of great interest.

Out of the various synthetic methodologies for the preparation of pyrazole derivatives available thus far, condensation of 1,3-dielectrophilic agents (typically 1,3-diketones or their synthetic equivalents) with hydrazines is considered the most versatile and commonly applied strategy [1,2,4,5]. However, this classical method often suffers from regioselectivity issues and leads to isomeric pyrazoles, along with other by-products, which require tedious separation, e.g., using chromatography techniques. Hence, (3 + 2)-cycloaddition processes are an attractive alternative and enable straightforward access to the pyrazole skeleton through simultaneous formation of new carbon–carbon and carbon–nitrogen bonds. In this context, diazoalkanes, and particularly nitrile imines, have been recognized as readily available and powerful 1,3-dipoles for the construction of the pyrazole ring [1,2,17].

On the other hand, the negative impacts on the environment and public health caused by the large amount of waste solvents produced during classical organic synthesis have to be taken into account. Recently, there has been a rapid development of green and sustainable synthetic protocols based on mechanochemical approaches, in which the reaction is activated by the absorption of mechanical energy originating from collisions of milling balls [18,19,20,21]. More importantly, these reactions can be performed either without any solvent or require only small amounts of so-called “liquid assisted grinding solvent” (LAGs), and in many instances the chemo- and regio-selectivity switch, leading to rather unexpected products being observed upon mechanochemical activation. Several interesting applications of mechanochemistry in the synthesis of pharmaceutically-relevant *N*-containing compounds, such as Dantrolene (muscle relaxant), Tolbutamide (antidiabetic), and Axitinib (anticancer), have been reported [22,23,24]. Furthermore, the presented technique has been successfully applied for preparation of pyrazoles, mainly via condensation reactions starting with 1,3-dicarbonyls [25,26,27,28,29,30], chalcones [31], or enaminones [32], and appropriate hydrazine derivatives. Notably, to the best of our knowledge, no mechanochemical nitrile imine (3 + 2)-cycloadditions leading to pyrazoles have been reported.

**Scheme 1:**
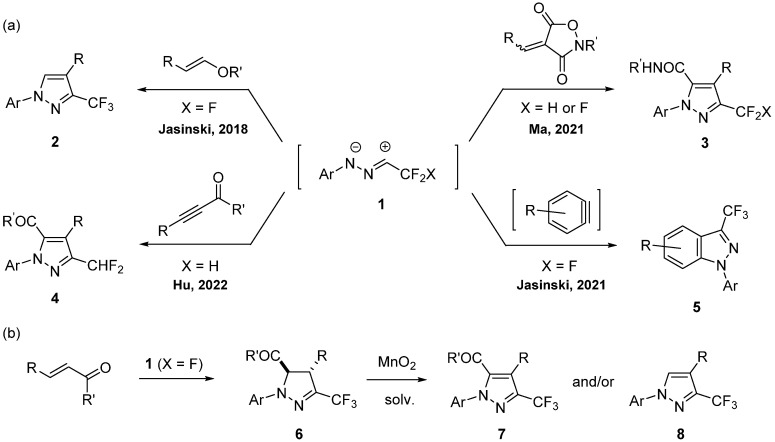
Synthesis of fluoroalkylated pyrazoles through: (**a**) (3 + 2)-cycloadditions of nitrile imines **1** with selected C=C or C≡C dipolarophiles, leading to monocyclic (**2**–**4**) [34,39,41] and bicyclic (**5**) [36] derivatives and (**b**) trapping of **1** with enones, followed by MnO_2_-mediated oxidation of the first 5-acylpyrazolines **6** formed, leading to polysubstituted 3-trifluoromethylpyrazoles **7** and/or **8**.

In a series of recent works, we and other groups have demonstrated fluorinated nitrile imines of type **1** and C=C or C≡C dipolarophiles as superior reaction partners for the efficient preparation of fluoroalkylated pyrazole and pyrazoline derivatives. For example, electron-rich enamines [33], vinyl ethers [34], alkoxyallenes [35], and benzynes [36], as well as electron-deficient nitro- [37] and cyanoalkenes [38], isoxazolidinediones [39], quinones [40], and ynone derivatives [41] have served as dipolarophilic agents. Exemplary reactions leading to polysubstituted pyrazoles **2**–**4** and bicyclic analogues **5** (indazoles) are depicted in Scheme 1a. More recently, we disclosed a general two-step protocol for two types of multi-substituted 3-trifluoromethylpyrazoles comprising (3 + 2)-cycloaddition of in situ generated nitrile imines **1** with chalcones, followed by MnO_2_-mediated aromatization of the first 5-acylpyrazolines **6** formed [42]. Remarkably, depending on the solvent used, the oxidation step preferentially afforded fully substituted pyrazoles **7** (in polar solvents such as DMF or DMSO) or proceeded via a deacylative pathway (in non-polar solvents, e.g., in hexane), leading to 1,3,4-trisubstituted pyrazoles **8** as major products (Scheme 1b). Taking into account the well-documented significance of both fluorinated and non-fluorinated pyrazoles in medicine and material sciences, the solvent-free mechanochemical protocols of the above (3 + 2)-cycloaddition reaction and subsequent oxidation step should be examined. Furthermore, the scope of the studied processes, towards non-fluorinated analogues, should also be checked. Here, we report our recent results on a two-step synthesis of 1,4-diarylpyrazoles functionalized with CF_3_, COOEt, Ac, or Ph groups at C(3) of the heterocyclic ring, under solvent-free ball-milling mechanochemical conditions.

## 2. Results and Discussion

**Scheme 2:**
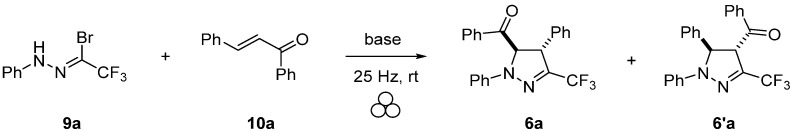
Base-catalyzed mechanochemical (3 + 2)-cycloaddition of trifluoromethylated nitrile imine **1a** derived from hydrazonoyl bromide **9a** and chalcone (**10a**), leading to the isomeric benzoyl-pyrazolines **6a** (major) and **6′a** (minor).

The required CF_3_-nitrile imines of type **1** are readily available propargyl-type 1,3-dipoles, which can be generated in situ via base-induced dehydrohalogenation of the respective hydrazonoyl halides (or pseudohalides) [17,43]. A series of key precursors, namely hydrazonoyl bromides **9**, were prepared according to the general literature protocols, starting with commercially available substrates, i.e., fluoral hydrate and arylhydrazines [43,44,45,46]. According to our previous observations, the reversible generation of trifluoroacetonitrile imines **1** from the corresponding bromide **9** proceeds smoothly upon treatment with excess Et_3_N, at room temperature, in anhydrous THF as the solvent of choice. For this reason, initial mechanochemical experiments (steel balls, ø 7 mm; 25 Hz) were carried out using the known *C*-trifluoromethyl-*N*-phenyl nitrile imine (**1a**) and chalcone (**10a**) selected as model substrates, in the presence of Et_3_N (Scheme 2). As evidenced by TLC monitoring, a rapid (3 + 2)-cycloaddition reaction was observed, and after 1 h the expected 3-trifluoromethylpyrazoline **6a** was identified as a major component of the crude reaction mixture, along with small amounts of regioisomeric derivative **6′a** (in ca. 7:1 ratio, respectively), however, in moderate yield (56% conversion estimated based on ^1^H NMR spectrum of crude mixture), as unconsumed chalcone **10a** accompanied by unidentified decomposition products of bromide **9a** were also detected. Then, the influence of a series of inorganic bases on the reaction course was briefly checked (Table 1). Whereas application of K_2_CO_3_ as a base enhanced the conversion significantly (82%), further optimization with respect to the amount of nitrile imine precursor **9a** (1.2 equiv.) and with the volume of the vessel used (5 mL) provided the (3 + 2)-cycloadducts in an excellent 93% yield. Subsequent separation by column chromatography provided spectroscopically pure samples of two pyrazolines, **6a** (75%) and **6′a** (13%). The relative orientation of substituents along the C(4)-C(5) bond in **6a** and **6′a** was established based on the ^1^H NMR spectra and by comparison with the literature data on other *trans*-configured 5-acylpyrazolines [42,47]. For example, in the case of compound **6a**, the diagnostic protons appeared as doublet of quartets (*J*_H-H_ = 5.6 Hz, ^4^*J*_H-F_ ≈ 0.9 Hz) at *δ* 4.37 (4-H) and as doublet (*J*_H-H_ = 5.6 Hz) at *δ* 5.76 (5-H), thereby confirming the fully diastereoselective addition of 1,3-dipole **1a** onto the C=C bond of the conjugated system of **10a**. The structure of minor isomer **6′a** was elucidated on the basis of ^1^H and ^13^C NMR supplemented with 2D NMR measurements (HMQC, HMBC). For example, in the ^1^H NMR spectrum of **6′a**, along with the characteristic set of signals attributed to phenyl groups, two additional absorptions, i.e., broadened doublet (*J* = 7.3 Hz) located at *δ* 5.04 (4-H) and doublet (*J*_H-H_ = 7.3 Hz) at *δ* 5.65 (5-H) nicely matched the proposed structure of **6′a**. Furthermore, in the ^13^C NMR spectrum of **6′a**, two diagnostic quartets found at *δ* 120.9 (^1^*J*_C-F_ = 269.8 Hz) and *δ* 133.5 (^2^*J*_C-F_ = 38.0 Hz), attributed to the CF_3_ group and C-3 atom, respectively, as well as a low intensity absorption (s) at *δ* 194.5 attributed to the C=O group, were found.

It should be noted that the reaction of **9a** with **10a** carried out under classical conditions, i.e., in THF solution at room temperature, leads to pyrazoline **6a** (79%) exclusively, although after a rather long reaction time (4 days) [42]. In contrast, the mechanochemical activation of the studied (3 + 2)-cycloaddition provided the desired material **6a** in a comparable yield (75%) after a remarkably shorter reaction time of 3 h, but the competitive formation of small amounts of isomeric product **6′a** was observed.

**Scheme 3:**
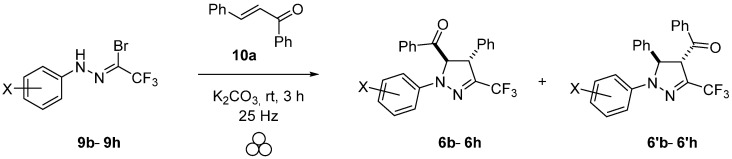
Mechanochemical synthesis of 5-benzoyl-4-phenyl-3-trifluoromethylpyrazolines **6b**–**6h** derived from chalcone (**10a**); scope of hydrazonoyl bromides **9**.

With the optimized conditions in hand, we next turned our attention to the scope and limitations of the developed mechanochemical 1,3-dipolar cycloaddition. A series of nitrile imine precursors of type **9**, bearing either electron-donating (**9b**–**9d**) or electron-withdrawing (**9e**, **9g**, and **9h**) groups X located at *para* position of the phenyl ring, as well as disubstituted derivative **9f** (2,4-Cl_2_), were examined in (3 + 2)-cycloadditions with a model chalcone (**10a**) (Scheme 3). As shown in Table 2, higher chemical yields were observed for reactions carried out with nitrile imine precursors **9b**–**9d***,* i.e., bearing groups increasing the electron density at the negatively charged *N*-termini of the in situ generated dipole **1**, and the expected products **6b**–**6d** (58–71%) were obtained after 3 h of only ball-milling. In contrast, in experiments performed with bromides functionalized with a strong EWG group (NO_2_, **9g**), and also with a PhCOO moiety (**9h**), complete consumption of the starting materials was observed after remarkably longer time (up to 24 h). In the latter cases, the formation of complex reaction mixtures also made the chromatographic isolation of the desired 5-benzoylpyrazolines **6** more difficult. Interestingly, despite the above differences, no remarkable impact of the electronic character of groups X on the regioselectivity of the studied (3 + 2)-cycloaddition could be observed. In all the cases, a mixture of isomeric products **6a**–**6h** and **6′a**–**6′h** in comparable ratios of ca. 4:1, respectively, were formed.

**Scheme 4:**
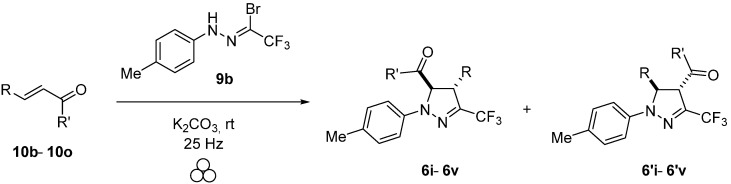
Synthesis of 5-acyl-3-trifluoromethylpyrazolines **6i**–**6v** using *N*-(*p*-tolyl) nitrile imine **1b** generated in situ from hydrazonoyl bromide **9b**; scope of chalcones **10**.

Next, to check the scope of chalcones and to test the functional group tolerance of mechanochemical (3 + 2)-cycloaddition, a series of aryl- and ferrocenyl-functionalized enones **10b**–**10o** were also added to the study and examined in reaction with *N*-(*p*-tolyl) nitrile imine **1b**, selected as a handful ^1^H NMR-diagnostic representative (Scheme 4). In general, the expected 5-acylpyrazolines **6i**–**6v** were obtained in moderate to high yields, although longer reaction times were required to lead the reaction to completion in most cases (Table 3). Thus, apart from halogens (Cl, Br) and haloalkyl units (additional CF_3_ group at phenyl ring), alkylamino and alkoxy substituents, as well as a ferrocenyl moiety, could be introduced.

Similarly to the results collected for series **6/6′a**–**6/6′h** (Scheme 2 and Scheme 3, Table 2), (3 + 2)-cycloadditions of **1b** with selected chalcones **10b**–**10o** proceeded in a comparable regioselectivity of ca. 4:1 in favor of 5-acylpyrazolines **6**. Again, only *trans*-configured products could be detected in the mother liquors. Interestingly, in the case of 3,4-methylenedioxy-functionalized chalcone (**10f**) and 3,4-dimethoxy analogue (**10e**), exceptionally high selectivity (ca. 9:1) or exclusive formation of target 5-acylpyrazolines **6m** and **6l**, respectively, was observed. On the other hand, the reaction of **1b** with another electron-rich chalcone, namely 4-(dimethylamino)chalcone (**10g**), provided only the expected (3 + 2)-cycloadducts **6n** and **6′n** as a ca. 2:1 mixture. Possibly, the observed decrease of selectivity resulted from the presence of the basic Me_2_N group in **10g**, which can compete with K_2_CO_3_ in dehydrohalogenation of **9b**, thereby changing the electronic properties of chalcone **10g**, due to protonation. The observed moderate yield in cycloadditions of **1b** with chalcones **10c** and **10m**, leading to pyrazolines **6j** (38%) and **6t** (39%), also deserves a brief comment. Seemingly, the presence of the redox-active Fc group alters the reaction outcome and leads to complex mixtures, irrespective of the substitution pattern in chalcone.

**Scheme 5:**
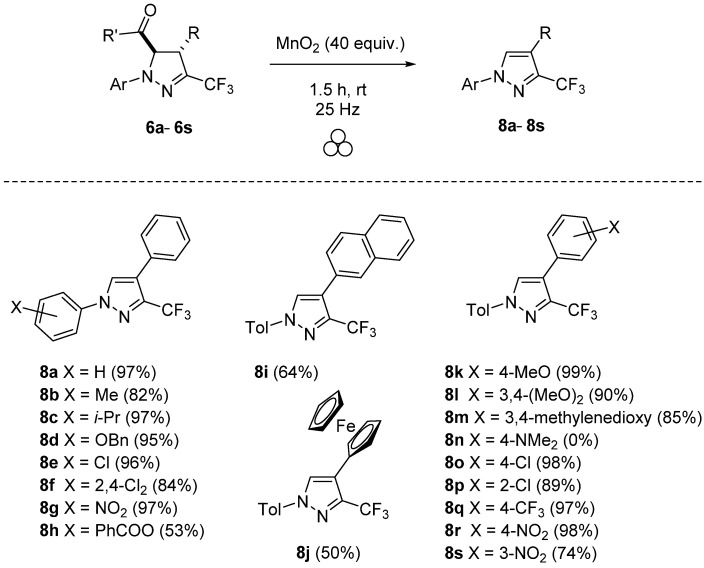
Mechanochemical MnO_2_-mediated deacylative oxidation of 5-acylpyrazolines **6a**–**6s**, leading to 1,4-diaryl-3-trifluoromethylpyrazoles **8a**–**8s**.

Prompted by the results disclosed in our recent work on the solvent-dependent oxidation of 5-benzoylpyrazolines [42], a series of 3-trifluoromethylated cycloadducts of type **6** were oxidized with an excess of activated MnO_2_ under mechanochemical conditions. In a typical experiment, pyrazoline **6a** (1.0 mmol) was reacted with oxidant (activated MnO_2_, ca. 85%, <10 μm, 40 equiv.) using zirconium oxide ball-milling equipment (ball, ø 10 mm; jar, 10 mL), at 25 Hz. After the reaction was complete (1.5 h), the resulting material was washed with AcOEt and filtered through a short silica gel pad, to give 1,5-diphenyl-3-trifluoromethylpyrazole (**8a**), isolated as a sole product in excellent purity and a yield of 97% (Scheme 5). The observed result for MnO_2_-mediated mechanochemical deacylative oxidation nicely correspond to the recently reported aromatizative debenzoylation of **6a** carried out in non-polar solvents (i.e., hexane solutions). However, the latter protocol provided the final product **8a** after 2 days, by heating the reactants in organic medium at 60 °C [42].

Unfortunately, an attempted one-pot two-step synthesis of pyrazole **8a** was in vain. In the mentioned experiment, hydrazonoyl bromide **9a** and chalcone **10a** were mechanochemically reacted under the developed conditions (in the presence of K_2_CO_3_), followed by treatment of the resulting crude reaction mixture with excess activated MnO_2_. To our surprise, none of the expected pyrazole **8a** was detected in the mixture, thus indicating the necessity of (at least partial) pre-purification of the intermediate 5-benzoylpyrazoline **6a**. Indeed, simple filtration of crude **6a** through a short silica gel pad enabled fast synthesis of desired material **8a**, which was isolated in a high 66% overall yield (for two steps).

**Scheme 6:**
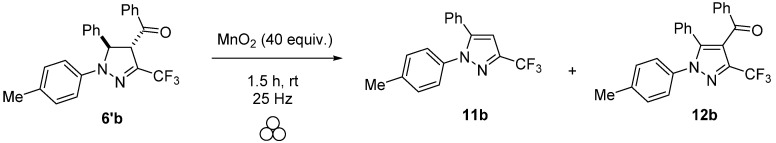
Synthesis of pyrazoles **11b** and **12b** formed via competitive deacylative vs. dehydrogenative aromatization of 4-benzoylpyrazoline **6′b**.

In continuation, a series of pyrazolines **6b**–**6s** was examined in reaction with MnO_2_ under mechanochemical activation to afford the expected 1,4-diaryl-3-trifluoromethylpyrazoles **8b**–**8s** identified as the exclusive aromatization products, which were generally isolated in excellent yields. Only in the case of 4-benzoyloxy derivative (**6h**) and ferrocenyl-functionalized analogue (**6j**), either partial decomposition of the starting material or competitive dehydrogenative oxidation, leading to a fully substituted analogue (**7j**), respectively, was observed, and moderate amounts of the final pyrazoles **8h** (53%) and **8j** (50%) were isolated. Surprisingly, the attempted oxidation of pyrazoline **6n** bearing Me_2_N group resulted in complete decomposition of the starting material under the applied conditions. In order to check the reaction outcome in mechanochemical oxidation of isomeric 4-acyl-pyrazolines of type **6′**, available as minor products in (3 + 2)-cycloaddition of nitrile imines **1** and chalcones **10**, a model *trans*-4-benzoyl-5-phenyl-1-*p*-tolyl-3-trifluoromethylpyrazoline (**6′b**) was also examined under analogous reaction conditions. As shown in Scheme 6, treatment of the starting material **6′b** with excess MnO_2_ provided, after 1.5 h of milling, a mixture of two pyrazole-based products in ca. 2:3 ratio, and they were identified as 5-phenyl-1-*p*-tolyl-3-trfiluoromethylpyrazole (**11b**, 38%) and its 4-benzoylated analogue **12b** (56%). This result indicates that, in contrast to 5-acylpyrazoline **6b**, ball-milling oxidation of its structural isomer 4-acylpyrazoline **6′b** proceeds in low chemoselectivity and leads to dehydrogenative oxidation of product **12b** as a major component of the mixture.

**Scheme 7:**
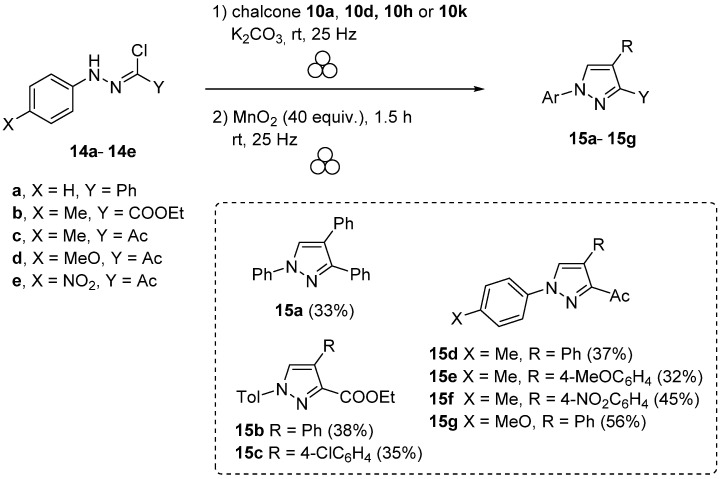
Two-step synthesis of pyrazoles **15a**–**15g** using non-fluorinated nitrile imine precursors of type **14**.

Finally, to further check the scope, a series of non-fluorinated pyrazolines **13a**–**13g** were prepared and examined in a mechanochemical oxidation reaction with activated MnO_2_. Following the general protocol, five nitrile imine precursors **14a**–**14e** bearing either phenyl group or selected electron-withdrawing substituents (COOEt, Ac) located at the *C*-termini were reacted with a set of representative chalcones: **10a** (X = H), **10d** (OMe), **10h** (Cl), and **10k** (X = NO_2_) (Scheme 7). The first formed 5-acylpyrazoline derivatives **13** were pre-purified by filtration through a short silica gel pad and subsequently reacted with MnO_2_ to provide the expected 1,3,4-trisubstituted pyrazoles **15a**–**15g** in an acceptable overall yield of 32–56% (for two steps). However, in the case of the highly electron-deficient nitrile imine **1e** functionalized with O_2_NC_6_H_4_- and Ac groups, the (3 + 2)-cycloaddition step with chalcone **10a** afforded a complex mixture in which trace amounts of the expected pyrazoline **13h** (<5%) were detected. The presented results indicate that, along with trifluoromethylated nitrile imines, analogues bearing aryl, ester or acyl groups can also be applied in the developed two-step synthesis of 1,3,4-trisubstituted pyrazoles.

**Scheme 8:**
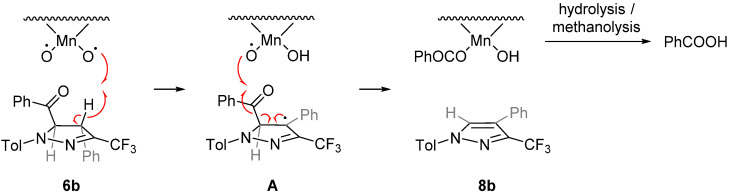
Proposed mechanism of deacylative oxidation of 5-acylpyrazolines.

It should be pointed out that all the presented deacylative oxidations of benzoyl-pyrazolines were performed using activated MnO_2_ (≈85% purity, <10 μm, Sigma Aldrich, St. Louis, MO, USA), which was used as received. In order to gain a greater insight about the studied transformation, non-activated manganese dioxide (Reagent Plus ^®^, >99%, Sigma Aldrich) was also tested, but in this case no deacylative aromatization could be observed when using 5-benzoylpyrazoline **6b** as a model compound. To test if hydroxyl radicals were involved in deacylative aromatization of **6b**, the latter experiment was repeated in the presence of trace amounts of water, but the reaction was not triggered. Finally, treatment of the resulting insoluble material formed in deacylative oxidation of **6b** with aqueous methanol released a colorless byproduct identified as benzoic acid. Based on these observations, the mechanism of the studied reaction is tentatively proposed. As depicted in Scheme 8, oxidation of **6b** proceeds preferentially at C(4), leading to fairly stable benzyl-type radical **A**. Then, the acyl group is transferred [48] from **A** onto the activated surface of the heterogeneous oxidant to give the aromatized product **8b** [49]. On the other hand, the presence of the benzoyl group at C(4) in isomeric pyrazoline **6′b** enhances the acidity of this position; and thus, the oxidation may possibly be initiated either at C(4) or at C(5), leading to a mixture of products formed via competitive dehydrogenation vs. deacylative aromatization processes.

## 3. Materials and Methods

### 3.1. Chemical Synthesis General Methods

**Experimental procedures:** The ball-milling apparatus used was a Retsch MM 400 mixer mill (Retsch GmbH, Haan, Germany). Mechanochemical (3 + 2)-cycloadditions were performed in 5 mL stainless steel jars, with three stainless steel balls (7 mm diameter); oxidation reactions were conducted in 10 mL zirconium oxide jars, with one zirconium oxide ball (10 mm diameter). Solvents (hexane, CH_2_Cl_2_, AcOEt) were purchased and used as received. Products were purified by filtration through a short silica gel plug or by standard column chromatography (CC) on silica gel (230–400 mesh; Merck, Kenilworth, NJ, USA). The NMR spectra were taken on a Bruker AVIII instrument (^1^H at 600 MHz, ^13^C at 151 MHz, and ^19^F at 565 MHz) (Bruker BioSpin AG, Fällanden, Switzerland). Chemical shifts are reported relative to solvent residual peaks; for CDCl_3_: ^1^H NMR: δ = 7.26, ^13^C NMR: δ = 77.16, or to CFCl_3_ (^19^F NMR: δ = 0.00) used as an external standard. Multiplicity of the signals in ^13^C NMR spectra were deduced based on supplementary 2D measurements (HMQC, HMBC). The IR spectra were measured with an Agilent Cary 630 FTIR spectrometer (Agilent Technologies, Santa Clara, CA, USA), in neat. MS (ESI) were performed with a Varian 500-MS LC Ion Trap (Varian, Inc., Walnut Creek, CA, USA), while high resolution MS (ESI-TOF) measurements were taken with a Waters Synapt G2-Si mass spectrometer (Waters Corporation, Milford, MA, USA). Elemental analyses were performed with a Vario EL III (Elementar Analysensysteme GmbH, Langenselbold, Germany) instrument. Melting points were determined in capillaries with a MEL-TEMP apparatus (Laboratory Devices, Holliston, MA, USA) and are uncorrected. ^1^H, ^13^C, and ^19^F NMR spectra of all new compounds can be found at Supplementary Materials file.

**Starting materials:** The CF_3_-nitrile imine precursors of type **9** were prepared by bromination of the corresponding trifluoroacetaldehyde arylhydrazones with NBS, according to the general protocol [43]. The required fluoral hydrazones were synthesized following the general literature procedure by condensation of aqueous fluoral hydrate (~75% in H_2_O) with commercial arylhydrazines [46]. Non-fluorinated hydrazonoyl chlorides **14a**–**14e** were prepared as previously reported [44,45]. Chalcones **10** were purchased or prepared via classical Claisen–Schmidt condensation, starting with appropriate aldehydes and methyl ketones, in ethanol. Activated MnO_2_ (ca. 85%, <10 μm, Sigma-Aldrich, product no. 217646-100G), as well as the other commercially available solvents and starting materials, were purchased and used as received.

#### 3.1.1. General Procedure for Mechanochemical Synthesis of Pyrazolines **6**, **6′**, and **13**

Hydrazonoyl halide **9** or **14** (1.2 mmol), chalcone **10** (1.0 mmol), and solid K_2_CO_3_ (1.3 mmol, 179 mg) were placed in a 5 mL stainless steel grinding jar with three stainless steel balls (7 mm diameter). The jar was closed and ball-milled at 25 Hz until the starting chalcone was fully consumed. Then, CH_2_Cl_2_ (10 mL) was added, the precipitate was filtered off, washed with CH_2_Cl_2_ (2 × 10 mL), and the solvent was removed under vacuum. The crude product of type **6** or **13** was purified by standard column chromatography (CC) or pre-purified by flash column chromatography (FCC) on silica. The structures of known pyrazolines **6c**–**6k, 6o, 6q, 6r, 6t**–**6v** were confirmed based on ^1^H NMR spectra supplemented by ESI-MS measurements and by comparison with original samples [42]; the byproducts **6′c**–**6′v** were not isolated. In the case of non-fluorinated analogues, crude pyrazolines **13a,13d**–**13g** were pre-purified by FCC and used for the next step, without further purification.

*trans-5-Benzoyl-1,4-diphenyl-3-trifluoromethyl-4,5-dihydro-1H-pyrazole* (**6a**) [50]: light yellow solid, 296 mg (75%), mp 159–161 °C. ^1^H NMR (600 MHz, CDCl_3_) *δ* 4.37 (dq, ^4^*J*_H-F_ ≈ 0.9 Hz, *J*_H-H_ = 5.6 Hz, 1H, 4-H), 5.76 (d, *J*_H-H_ = 5.6 Hz, 1H, 5-H), 6.94–7.06, 7.19–7.29, 7.39–7.44, 7.48–7.52, 7.65–7.68, 7.87–7.89 (6m, 3H, 4H, 3H, 2H, 1H, 2H). ^13^C NMR (151 MHz, CDCl_3_) *δ* 55.6, 74.3, 113.8, 120.9 (q, ^1^*J*_C-F_ = 270.6 Hz, CF_3_), 121.6, 127.7, 128.9, 129.2, 129.3, 129.5, 129.7, 133.1, 134.7, 137.5, 138.1 (q, ^2^*J*_C-F_ = 37.0 Hz, C-3), 142.7, 192.1. ^19^F NMR (565 MHz, CDCl_3_) *δ* −63.0 (s_br_, CF_3_). ESI-MS (*m*/*z*) 417.2 (100, [M + Na]^+^).

*trans-4-Benzoyl-1,5-diphenyl-3-trifluoromethyl-4,5-dihydro-1H-pyrazole* (**6′a**): obtained as a minor product in the reaction of **9a** with **10a**; yellow solid, 51 mg (13%), mp 125–126 °C.

^1^H NMR (600 MHz, CDCl_3_) *δ* 5.04 (d_br_, *J*_H-H_ ≈ 7.3 Hz, 1H, 4-H), 5.65 (d, *J*_H-H_ = 7.3 Hz, 1H, 5-H), 6.88–6.91, 7.03–7.06, 7.17–7.20, 7.23–7.25, 7.33–7.40, 7.48–7.51, 7.63–7.66, 7.88–7.90 (8m, 1H, 2H, 2H, 2H, 3H, 2H, 1H, 2H). ^13^C NMR (151 MHz, CDCl_3_) *δ* 61.2, 71.0, 114.8, 120.9 (q, ^1^*J*_C-F_ = 269.8 Hz, CF_3_), 121.7, 126.0, 128.9, 129.14, 129.15, 129.16, 129.8, 133.5 (q, ^2^*J*_C-F_ = 38.0 Hz, C-3), 134.5, 135.5, 139.6, 142.6, 194.5. ^19^F NMR (565 MHz, CDCl_3_) *δ* −63.1 (s, CF_3_). IR (neat) *v* 1677, 1595, 1577, 1301, 1264, 1208, 1148, 1118, 1066 cm^−1^. ESI-MS (*m*/*z*) 417.1 (31, [M + Na]^+^), 395.2 (100, [M + H]^+^).

*trans-5-Benzoyl-1-(p-tolyl)-4-phenyl-3-trifluoromethyl-4,5-dihydro-1H-pyrazole* (**6b**) [51]: light yellow solid, 286 mg (70%), mp 145–147 °C. ^1^H NMR (600 MHz, CDCl_3_) *δ* 2.30 (s, 3H, Me), 4.38 (dq, ^4^*J*_H-F_ ≈ 1.0 Hz, *J*_H-H_ ≈ 5.7 Hz, 1H, 4-H), 5.78 (d_br_, *J* ≈ 5.7 Hz, 1H, 5-H), 6.97, 7.09 (2d, *J* = 8.6 Hz, 2H each), 7.21–7.25, 7.40–7.52, 7.66–7.69, 7.88–7.91 (4m, 2H, 5H, 1H, 2H). ^13^C NMR (151 MHz, CDCl_3_) *δ* 20.7, 55.6, 74.6, 113.9, 121.0 (q, ^1^*J*_C-F_ = 270.3 Hz, CF_3_), 127.5, 129.0, 129.1, 129.3, 129.7, 130.0, 130.9, 133.2, 134.6, 137.4 (q, ^2^*J*_C-F_ = 36.8 Hz, C-3), 137.6, 140.5, 192.3. ^19^F NMR (565 MHz, CDCl_3_) *δ* −63.1 (s, CF_3_). ESI-MS (*m*/*z*) 431.2 (100, [M + Na]^+^).

*trans-4-Benzoyl-1-(p-tolyl)-5-phenyl-3-trifluoromethyl-4,5-dihydro-1H-pyrazole* (**6′b**): obtained as a minor product in the reaction of **9b** with **10a**; thick yellow oil, 53 mg (13%). ^1^ H NMR (600 MHz, CDCl_3_) *δ* 2.23 (s, 3H, Me), 5.04 (dq_br_, ^4^*J*_H-F_ ≈ 1.6 Hz, *J*_H-H_ ≈ 7.5 Hz, 1H, 4-H), 5.64 (d, *J*_H-H_ ≈ 7.5 Hz, 1H, 5-H), 6.93–7.00, 7.22–7.25, 7.32–7.39, 7.47–7.50, 7.63–7.66, 7.87–7.90 (6m, 4H, 2H, 3H, 2H, 1H, 2H). ^13^C NMR (151 MHz, CDCl_3_) *δ* 20.7, 61.1, 71.2, 114.8, 121.0 (q, ^1^*J*_C-F_ = 269.5 Hz, CF_3_), 126.1, 128.8, 129.12, 129.14, 129.7, 129.8, 131.2, 132.8 (q, ^2^*J*_C-F_ = 37.9 Hz, C-3), 134.5, 135.4, 139.7, 140.3, 194.6. ^19^F NMR (565 MHz, CDCl_3_) *δ* −63.0 (s, CF_3_). IR (neat) *v* 1752, 1662, 1495, 1446, 1260, 1219, 1163, 1133 cm^−1^. ESI-MS (*m*/*z*) 431.4 (100, [M + Na]^+^), 409.5 (39, [M + H]^+^). Anal. Calcd for C_24_H_19_F_3_N_2_O (408.1): C 70.58, H 4.69, N 6.86; found: C 70.49, H 4.69, N 6.89.

*trans-5-Benzoyl-4-(3′,4′-dimethoxyphenyl)-1-(p-tolyl)-3-trifluoromethyl-4,5-dihydro-1H-pyrazole* (**6l**): light yellow solid, 347 mg (74%), mp 122–123 °C. ^1^H NMR (600 MHz, CDCl_3_) *δ* 2.27 (s, 3H, Me), 3.81, 3.91 (2s, 3H each, 2OMe), 4.33 (d_br_, *J* ≈ 5.9 Hz, 1H, 4-H), 5.73 (d, *J* = 5.9 Hz, 1H, 5-H), 6.65 (d, *J* = 2.1 Hz, 1H), 6.76 (dd, *J* = 2.1, 8.2 Hz, 1H), 6.88 (d, *J* = 8.2 Hz, 1 H), 6.91–6.93, 7.05–7.08, 7.48–7.51, 7.64–7.68, 7.87–7.90 (5m, 2H, 2H, 2H, 1H, 2H). ^13^C NMR (151 MHz, CDCl_3_) *δ* 20.7, 55.4, 56.1, 56.2, 74.5, 110.4, 111.8, 113.9, 120.2, 121.0 (q, ^1^*J*_C-F_ = 270.4 Hz, CF_3_), 129.3 *, 129.8, 130.0, 131.0, 133.2, 134.6, 137.4 (q, ^2^*J*_C-F_ = 36.6 Hz, C-3), 140.5, 149.5, 149.8, 192.4; * higher intensity. ^19^F NMR (565 MHz, CDCl_3_) *δ* −62.2 (s, CF_3_). IR (neat) *v* 1695, 1595, 1513, 1450, 1293, 1230, 1118 cm^−1^. HRMS (ESI-TOF) *m/z*: [M + H]^+^ calcd for C_26_H_24_F_3_N_2_O_3_ 469.1739, found 469.1743.

*trans-5-Benzoyl-4-(3′,4′-methylenedioxyphenyl)-1-(p-tolyl)-3-trifluoromethyl-4,5-dihydro-1H-pyrazole* (**6m**): pale yellow solid, 307 mg (68%), mp 125–126 °C. ^1^H NMR (600 MHz, CDCl_3_) *δ* 2.28 (s, 3H, Me), 4.31 (d_br_, *J* ≈ 5.5 Hz, 1H, 4-H), 5.73 (d, *J* = 5.5 Hz, 1H, 5-H), 6.01 (AB system, *J* = 4.8 Hz, 2H, OCH_2_O), 6.66–6.69 (m, 2H), 6.82 (d, *J* = 7.8 Hz, 1H), 6.92–6.94, 7.07–7.09, 7.50–7.54, 7.66–7.69, 7.90–7.92 (5m, 2H, 2H, 2H, 1H, 2H). ^13^C NMR (151 MHz, CDCl_3_) *δ* 20.7, 55.3, 74.4, 101.7, 107.6, 109.0, 113.8, 121.0 (q, ^1^*J*_C-F_ = 270.3 Hz, CF_3_), 121.9, 129.2, 129.3, 130.0, 131.0, 131.2, 133.1, 134.6, 137.5 (q, ^2^*J*_C-F_ = 36.7 Hz, C-3), 140.4, 148.2, 148.9, 192.2. ^19^F NMR (565 MHz, CDCl_3_) *δ* −62.3 (s, CF_3_). IR (neat) *v* 1685, 1595, 1517, 1446, 1245, 1118 cm^−1^. HRMS (ESI-TOF) *m/z*: [M + H]^+^ calcd for C_25_H_20_F_3_N_2_O_3_ 453.1426, found 453.1427.

*trans-5-Benzoyl-4-(4′-dimethylaminophenyl)-1-(p-tolyl)-3-trifluoromethyl-4,5-dihydro-1H-pyrazole* (**6n**): orange solid, 208 mg (46%), mp 163–165 °C. ^1^H NMR (600 MHz, CDCl_3_) *δ* 2.27 (s, 3H, Me), 2.99 (s, 6H, 2Me), 4.30 (d_br_, *J* ≈ 5.5 Hz, 1H, 4-H), 5.69 (d, *J* = 5.5 Hz, 1H, 5-H), 6.69–6.72, 6.90–6.92, 7.03–7.07, 7.47–7.51, 7.64–7.66, 7.89–7.91 (6m, 2H, 2H, 4H, 2H, 1H, 2H). ^13^C NMR (151 MHz, CDCl_3_) *δ* 20.7, 40.5, 55.1, 74.7, 112.9, 113.7, 121.1 (q, ^1^*J*_C-F_ = 270.5 Hz, CF_3_), 124.6, 128.5, 129.22, 129.25, 130.0, 130.6, 133.3, 134.4, 138.1 (q, ^2^*J*_C-F_ = 36.2 Hz, C-3), 140.7, 150.7, 192.5. ^19^F NMR (565 MHz, CDCl_3_) *δ* −62.3 (s, CF_3_). IR (neat) *v* 1696, 1595, 1517, 1297, 1230, 1185, 1118, 1066 cm^−1^. ESI-MS (*m*/*z*) 474.4 (100, [M + Na]^+^), 452.4 (97, [M + H]^+^). Anal. Calcd for C_26_H_24_F_3_N_3_O (451.2): C 69.17, H 5.36, N 9.31; found: C 69.11, H 5.26, N 9.30.

*trans-5-Benzoyl-4-(2′-chlorophenyl)-1-(p-tolyl)-3-trifluoromethyl-4,5-dihydro-1H-pyrazole* (**6p**): thick light orange oil, 252 mg (57%). ^1^H NMR (600 MHz, CDCl_3_) *δ* 2.28 (s, 3H, Me), 5.10 (s_br_, 1H, 4-H), 5.76 (s_br_, 1H, 5-H), 6.93–6.95, 7.06–7.09, 7.25–7.36, 7.43–7.50, 7.64–7.67, 7.84–7.88 (6m, 2H, 2H, 3H, 3H, 1H, 2H). ^13^C NMR (151 MHz, CDCl_3_) *δ* 20.7, 50.9(br), 74.0(br), 113.9, 120.8 (q, ^1^*J*_C-F_ = 270.3 Hz, CF_3_), 128.4(br), 129.1, 129.2 *, 130.0, 130.2, 130.4(br), 131.2, 133.2(br), 136.6, 135.3(br), 136.8 (q_br_, ^2^*J*_C-F_ ≈ 37.0 Hz, C-3), 140.4, 192.6; *higher intensity. ^19^F NMR (565 MHz, CDCl_3_) *δ* −62.6 (s, CF_3_). IR (neat) *v* 1692, 1599, 1517, 1297, 1230, 1118, 1066 cm^−1^. ESI-MS (*m*/*z*) 465.4 (100, [M + Na]^+^), 443.5 (83, [M + H]^+^). Anal. Calcd for C_24_H_18_F_3_N_2_O (442.1): C 65.09, H 4.10, N 6.33; found: C 65.00, H 4.02, N 6.14.

*trans-5-Benzoyl-4-(3′-nitrophenyl)-1-(p-tolyl)-3-trifluoromethyl-4,5-dihydro-1H-pyrazole* (**6s**): light yellow solid, 295 mg (65%), mp 170–172 °C. ^1^H NMR (600 MHz, CDCl_3_) *δ* 2.28 (s, 3H, Me), 4.50 (d_br_, *J* ≈ 5.6 Hz, 1H, 4-H), 5.75 (d, *J* = 5.6 Hz, 1H, 5-H), 6.93–6.95, 7.07–7.09, 7.50–7.54 (3m, 2H, 2H, 2H), 7.55 (dt, *J* = 1.4, 7.8 Hz, 1H), 7.62–7.65, 7.68–7.71, 7.85–7.87 (3m, 1H, 1H, 2H), 8.07 (pseudo-t, *J* ≈ 2.0 Hz, 1H), 8.28 (ddd, *J* = 1.1, 2.2, 8.2 Hz, 1 H). ^13^C NMR (151 MHz, CDCl_3_) *δ* 20.7, 54.8, 74.1, 114.1, 120.8 (q, ^1^*J*_C-F_ = 270.2 Hz, CF_3_), 122.7, 124.1, 129.1, 129.6, 130.1, 131.0, 131.7, 132.9, 133.6, 135.0, 136.1 (q, ^2^*J*_C-F_ = 37.3 Hz, C-3), 139.5, 140.0, 149.0, 191.6. ^19^F NMR (565 MHz, CDCl_3_) *δ* −62.2 (s, CF_3_). IR (neat) *v* 1689, 1595, 1536, 1353, 1297, 1230, 1152, 1122, 1070 cm^−1^. ESI-MS (*m*/*z*) 476.4 (100, [M + Na]^+^), 454.4 (50, [M + H]^+^). Anal. Calcd for C_24_H_18_F_3_N_3_O_3_ (453.1): C 63.58, H 4.00, N 9.27; found: C 63.49, H 4.04, N 9.29.

*Ethyl trans-5-benzoyl-4-phenyl-1-(p-tolyl)-4,5-dihydro-1H-pyrazole-3-carboxylate* (**13b**): light yellow solid, 234 mg (57%), mp 133–134 °C. ^1^H NMR (600 MHz, CDCl_3_) *δ* 1.20 (t, *J* = 7.1 Hz, 3H, Et), 2.28 (s, 3H, Me), 4.13 (dq, *J* = 7.1, 10.9 Hz, 1H, Et), 4.20 (dq, *J* = 7.1, 10.9 Hz, 1H, Et), 4.46 (d, *J* = 4.9 Hz, 1H, 4-H), 5.79 (d, *J* = 4.9 Hz, 1H, 5-H), 7.02–7.09, 7.21–7.23, 7.34–7.40, 7.49–7.52, 7.65–7.67, 7.90–7.92 (6m, 4H, 2H, 3H, 2H, 1H, 2H). ^13^C NMR (151 MHz, CDCl_3_) *δ* 14.2, 20.7, 55.4, 61.2, 74.3, 114.4, 127.5, 128.4, 129.2, 129.3, 129.5, 130.0, 131.4, 133.1, 134.5, 139.4, 139.98, 139.99, 161.8, 192.1. IR (neat) *v* 1696, 1513, 1279, 1219, 1152, 1100, 1014 cm^−1^. ESI-MS (*m*/*z*) 435.4 (100, [M + Na]^+^), 413.4 (31, [M + H]^+^). Anal. Calcd for C_26_H_24_N_2_O_3_ (412.2): C 75.71, H 5.86, N 6.79; found: C 75.71, H 6.04, N 6.80.

*Ethyl trans-5-benzoyl-4-(4′-chlorophenyl)-1-(p-tolyl)-4,5-dihydro-1H-pyrazole-3-carboxylate* (**13c**): yellow solid, 245 mg (55%), mp 157–159 °C. ^1^H NMR (600 MHz, CDCl_3_) *δ* 1.22 (t, *J* = 7.1 Hz, 3H, Et), 2.27 (s, 3H, Me), 4.14 (dq, *J* = 7.1, 10.9 Hz, 1H, Et), 4.21 (dq, *J* = 7.1, 10.9 Hz, 1H, Et), 4.42 (d, *J* = 5.0 Hz, 1H, 4-H), 5.74 (d, *J* = 5.0 Hz, 1H, 5-H), 7.00–7.03, 7.07–7.09, 7.14–7.16, 7.34–7.36, 7.49–7.53, 7.66–7.69, 7.87–7.89 (7m, 2H, 2H 2H, 2H, 2H, 1H, 2H). ^13^C NMR (151 MHz, CDCl_3_) *δ* 14.3, 20.8, 54.8, 61.3, 74.1, 114.4, 128.9, 129.1, 129.4, 129.7, 130.0, 131.7, 133.0, 134.4, 134.7, 138.0, 139.5, 139.8, 161.7, 191.8. ESI-MS (*m*/*z*) 469.4 (100, [M + Na]^+^), 447.4 (63, [M + H]^+^). Anal. Calcd for C_26_H_23_ClN_2_O_3_ (446.1): C 69.87, H 5.19, N 6.27; found: C 69.72, H 5.04, N 6.01.

#### 3.1.2. General Procedure for Oxidation Reactions with Activated Manganese Dioxide

5-Acylpyrazoline of type **6** or **13** (1.0 mmol) and activated MnO_2_ (40 mmol, 4.09 g) were placed in a 10 mL zirconium oxide grinding jar with one zirconium oxide ball (10 mm diameter). The jar was closed and subjected to grinding for 1.5 h in a vibratory ball-mill operated at 25Hz. After AcOEt (20 mL) was added, the resulting mixture was filtered through a thin pad of silica gel and the solvent was evaporated to give pyrazole **8** or **15**. In the case of 4-benzoylpyrazoline **6′b**, the resulting products **11b** and **12b** were purified using standard column chromatography (SiO_2_). The structure of known fluorinated pyrazoles, i.e., **8a**–**8k**, **8n**, **8q**, and **8r** were confirmed based on ^1^H NMR spectra and by comparison with the original samples [42].

*4-(3′,4′-Dimethoxyphenyl)-1-(p-tolyl)-3-trifluoromethylpyrazole* (**8l**): colorless solid, 326 mg (90%), mp 129–130 °C. ^1^H NMR (600 MHz, CDCl_3_) *δ* 2.38 (s, 3H, Me), 3.898, 3.901 (2s, 3H each, 2OMe), 6.88–6.91, 6.99–7.03, 7.25–7.27, 7.58–7.60 (4m, 1H, 2H, 2H, 2H), 7.93 (s, 1H, 5-H). ^13^C NMR (151 MHz, CDCl_3_) *δ* 21.0, 55.90, 55.91, 111.3, 112.0, 119.6, 120.9, 121.8 (q, ^1^*J*_C-F_ = 269.7 Hz, CF_3_), 122.9, 123.5(br), 127.3, 130.2, 137.0, 137.8, 139.9 (q, ^2^*J*_C-F_ = 36.4 Hz, C-3), 148.9*; *higher intensity. ^19^F NMR (565 MHz, CDCl_3_) *δ* −59.3 (s, CF_3_). IR (neat) *v* 1491, 1241, 1163, 1118 cm^−1^. (–)-ESI-MS (*m*/*z*) 361.4 (100, [M–H]^–^). Anal. Calcd for C_19_H_17_F_3_N_2_O_2_ (362.1): C 62.98, H 4.73, N 7.73; found: C 63.00, H 4.69, N 7.44.

*4-(3′,4′-Methylenedioxyphenyl)-1-(p-tolyl)-3-trifluoromethylpyrazole* (**8m**): colorless solid, 294 mg (85%), mp 99–100 °C. ^1^H NMR (600 MHz, CDCl_3_) *δ* 2.40 (s, 3H, Me), 6.00 (s, 2H, OCH_2_O), 6.85–6.87, 6.93–6.95, 7.27–7.29, 7.59–7.61 (4m, 1H, 2H, 2H, 2H), 7.90 (s_br_, 1H, 5-H). ^13^C NMR (151 MHz, CDCl_3_) *δ* 21.1, 101.4, 108.5, 109.3, 119.7, 121.7 (q, ^1^*J*_C-F_ = 269.8 Hz, CF_3_), 122.4(br), 123.4(br), 127.5, 130.2, 137.1, 137.9, 140.0 (q, ^2^*J*_C-F_ = 36.5 Hz, C-3), 147.6, 147.9. ^19^F NMR (565 MHz, CDCl_3_) *δ* −59.4 (s, CF_3_). IR (neat) *v* 1480, 1223, 1167, 1118, 1036 cm^−1^. ESI-MS (*m*/*z*) 369.4 (100, [M + Na]^+^), 347.4 (76, [M + H]^+^). Anal. Calcd for C_18_H_13_F_3_N_2_O_2_ (346.1): C 62.43, H 3.78, N 8.09; found: C 62.60, H 3.92, N 8.08.

*4-(2′-Chlorophenyl)-1-(p-tolyl)-3-trifluoromethylpyrazole* (**8p**): thick light yellow oil, 299 mg (89%). ^1^H NMR (600 MHz, CDCl_3_) *δ* 2.42 (s, 3H, Me), 7.29–7.36, 7.40–7.42, 7.48–7.51, 7.62–7.65 (4m, 4H, 1H, 1H, 2H), 7.99 (s, 1H, 5-H). ^13^C NMR (151 MHz, CDCl_3_) *δ* 21.1, 119.7(br), 119.8, 121.4 (q, ^1^*J*_C-F_ = 270.1 Hz, CF_3_), 126.7, 128.8, 129.5, 129.7, 129.8, 130.3, 132.2(br), 134.1, 137.1, 138.0, 141.3 (q, ^2^*J*_C-F_ = 36.6 Hz, C-3). ^19^F NMR (565 MHz, CDCl_3_) *δ* −60.0 (s, CF_3_). IR (neat) *v* 1521, 1495, 1290, 1223, 1170, 1116, 1062 cm^−1^. ESI-MS (*m*/*z*) 359.3 (23, [M + Na]^+^), 337.3 (100, [M + H]^+^). Anal. Calcd for C_17_H_12_ClF_3_N_2_ (336.1): C 60.64, H 3.59, N 8.32; found: C 60.51, H 3.39, N 8.47.

*4-(3′-Nitrophenyl)-1-(p-tolyl)-3-trifluoromethylpyrazole* (**8s**): colorless solid, 257 mg (74%), mp 147–148 °C. ^1^H NMR (600 MHz, CDCl_3_) *δ* 2.43 (s, 3H, Me), 7.31–7.33, 7.61–7.64, 7.82–7.84 (3m, 2H, 3H, 1H), 8.08 (s, 1H, 5-H), 8.24 (ddd, *J* = 1.0, 2.3, 8.2 Hz, 1H), 8.34 (pseudo-t, *J* ≈ 2.0 Hz, 1H). ^13^C NMR (151 MHz, CDCl_3_) *δ* 21.2, 119.9, 121.3(br), 121.5 (q, ^1^*J*_C-F_ = 269.8 Hz, CF_3_), 122.9, 123.6, 128.0, 129.8, 130.4, 132.2, 134.8(br), 136.8, 138.5, 140.2 (q, ^2^*J*_C-F_ = 37.1 Hz, C-3), 148.5. ^19^F NMR (565 MHz, CDCl_3_) *δ* −59.4 (s, CF_3_). IR (neat) *v* 1521, 1349, 1282, 1226, 1170, 1118, 1074 cm^−1^. ESI-MS (*m*/*z*) 370.3 (100, [M + Na]^+^), 348.3 (70, [M + H]^+^). Anal. Calcd for C_17_H_12_F_3_N_3_O_2_ (347.1): C 58.79, H 3.48, N 12.10; found: C 58.85, H 3.51, N 12.08.

*5-Phenyl-1-(p-tolyl)-3-trifluoromethylpyrazole* (**11b**) [34]: obtained as a minor product in oxidation of **6′b**; light yellow solid, 114 mg (38%), mp 74–76 °C. ^1^H NMR (600 MHz, CDCl_3_) *δ* 2.37 (s, 3H, Me), 6.74 (s_br_, 1H, 4-H), 7.14–7.24, 7.30–7.36 (2m, 6H, 3H). ^13^C NMR (151 MHz, CDCl_3_) *δ* 21.3, 105.5, 121.5 (q, ^1^*J*_C-F_ = 268.8 Hz, CF_3_), 125.5, 128.8, 128.95, 129.02, 129.5(br), 129.8, 137.0, 138.6, 143.2 (q, ^2^*J*_C-F_ = 38.3 Hz, C-3), 144.7. ^19^F NMR (565 MHz, CDCl_3_) *δ* −62.2 (s, CF_3_). IR (neat) *v* 1454, 1230, 1129, 1073 cm^−1^. HRMS (ESI-TOF) *m/z*: [M + H]^+^ calcd for C_17_H_14_F_3_N_2_ 303.1109, found 303.1104.

*4-Benzoyl-5-phenyl-1-(p-tolyl)-3-trifluoromethylpyrazole* (**12b**): colorless solid, 228 mg (56%), mp 139–140 °C. ^1^H NMR (600 MHz, CDCl_3_) *δ* 2.35 (s, 3H, Me), 7.07–7.10, 7.13–7.21, 7.28–7.32, 7.43–7.46, 7.72–7.74 (5m, 2H, 7H, 2H, 1H, 2H). ^13^C NMR (151 MHz, CDCl_3_) *δ* 21.3, 119.8(br), 121.0 (q, ^1^*J*_C-F_ = 270.4 Hz, CF_3_), 125.4, 127.9, 128.4, 128.7, 129.5, 129.8, 129.9, 130.1, 133.5, 136.3, 137.5, 139.0, 141.5 (q, ^2^*J*_C-F_ = 37.9 Hz, C-3), 144.4. ^19^F NMR (565 MHz, CDCl_3_) *δ* −60.3 (s, CF_3_). IR (neat) *v* 1659, 1484, 1443, 1223, 1156, 1129, 1059 cm^−1^. HRMS (ESI-TOF) *m/z*: [M + H]^+^ calcd for C_24_H_18_F_3_N_2_O 407.1371, found 407.1369.

*1,3,4-Triphenylpyrazole* (**15a**) [52]: light yellow solid, 97 mg (33%; for two steps, starting with 1.0 mmol of chalcone **10a** and chloride **14a**), mp 96–98 °C. ^1^H NMR (600 MHz, CDCl_3_) *δ* 7.29–7.37, 7.47–7.50, 7.60–7.62, 7.80–7.82 (4m, 9H, 2H, 2H, 2H), 8.03 (s, 1H, 5-H). ^13^C NMR (151 MHz, CDCl_3_) *δ* 119.1, 123.1, 126.6, 126.8, 127.1, 128.1, 128.5, 128.6, 128.7, 128.9, 129.6, 133.0, 133.3, 140.1, 150.6. IR (neat) *v* 1722, 1599, 1502, 1401, 1215, 1059 cm^−1^. ESI-MS (*m*/*z*) 297.3 (100, [M + H]^+^).

*Ethyl 4-phenyl-1-(p-tolyl)-pyrazole-3-carboxylate* (**15b**): colorless solid, 205 mg (67%), mp 99–102 °C. ^1^H NMR (600 MHz, CDCl_3_) *δ* 1.32 (t, *J* = 7.1 Hz, 3H, Et), 2.41 (s, 3H, Me), 4.37 (q, *J* = 7.1 Hz, 2H, Et), 7.27–7.29, 7.33–7.37, 7.39–7.42, 7.51–7.53, 7.64–7.66 (5m, 2H, 1H, 2H, 2H, 2H), 7.93 (s, 1H, 5-H). ^13^C NMR (151 MHz, CDCl_3_) *δ* 14.3, 21.2, 61.2, 120.1, 127.5, 127.7, 127.8, 128.2, 129.5, 130.2, 131.7, 137.4, 137.8, 141.2, 162.7. IR (neat) *v* 1722, 1610, 1517, 1465, 1279, 1226, 1141 cm^−1^. ESI-MS (*m*/*z*) 329.2 (25, [M + Na]^+^), 307.2 (100, [M + H]^+^). Anal. Calcd for C_19_H_18_N_2_O_2_ (306.1): C 74.49, H 5.92, N 9.14; found: C 74.47, H 6.00, N 9.21.

*Ethyl 4-(4′-chlorophenyl)-1-(p-tolyl)-pyrazole-3-carboxylate* (**15c**): colorless solid, 218 mg (64%), mp 136–137 °C. ^1^H NMR (600 MHz, CDCl_3_) *δ* 1.34 (t, *J* = 7.1 Hz, 3H, Et), 2.41 (s, 3H, Me), 4.38 (q, *J* = 7.1 Hz, 2H, Et), 7.27–7.29, 7.36–7.38, 7.45–7.47, 7.63–7.66 (4m, 2H, 2H, 2H, 2H), 7.92 (s, 1H, 5-H). ^13^C NMR (151 MHz, CDCl_3_) *δ* 14.4, 21.2, 61.3, 120.1, 126.4, 127.8, 128.4, 130.18, 130.20, 133.7, 137.2, 138.0, 141.1, 162.5. IR (neat) *v* 1707, 1476, 1442, 1349, 1282, 1226, 1156, 1107, 1077, 1033 cm^−1^. HRMS (ESI-TOF) *m/z*: [M + H]^+^ calcd for C_19_H_18_ClN_2_O_2_ 341.1057, found 341.1063.

*3-Acetyl-4-phenyl-1-(p-tolyl)-pyrazole* (**15d**): colorless solid, 102 mg (37%; for two steps, starting with 1.0 mmol of chalcone **10a** and chloride **14c**), mp 137–139 °C. ^1^H NMR (600 MHz, CDCl_3_) *δ* 2.42 (s, 3H, Me), 2.69 (s, 3H, Ac), 7.29–7.35, 7.38–7.41, 7.55–7.57, 7.65–7.67 (4m, 3H, 2H, 2H, 2H), 7.94 (s, 1H, 5-H). ^13^C NMR (151 MHz, CDCl_3_) *δ* 21.2, 28.1, 119.6, 126.4, 127.7, 127.9, 128.3, 129.4, 130.3, 131.7, 137.4, 137.8, 137.6, 194.8. IR (neat) *v* 1681, 1517, 1349, 1219, 1111 cm^−1^. ESI-MS (*m*/*z*) 299.3 (100, [M + Na]^+^), 277.3 (87, [M + H]^+^). Anal. Calcd for C_18_H_16_N_2_O (276.1): C 78.24, H 5.84, N 10.14; found: C 78.01, H 5.82, N 10.00.

*3-Acetyl-4-(4′-methoxyphenyl)-1-(p-tolyl)-pyrazole* (**15e**): light brown solid, 98 mg (32%; for two steps, starting with 1.0 mmol of chalcone **10d** and chloride **14c**), mp 108–109 °C. ^1^H NMR (600 MHz, CDCl_3_) *δ* 2.42 (s, 3H, Me), 2.68 (s, 3H, Ac), 3.84 (s, 3H, OMe), 6.92–6.95, 7.29–7.32, 7.49–7.52, 7.64–7.67 (4m, 2H each), 7.89 (s, 1H, 5-H). ^13^C NMR (151 MHz, CDCl_3_) *δ* 21.2, 28.1, 55.5, 113.8, 119.6, 124.1, 126.1, 127.5, 130.3, 130.6, 137.5, 137.7, 147.6, 159.3, 194.8. IR (neat) *v* 1692, 1551, 1498, 1450, 1387, 1346, 1249, 1182, 1107, 1029 cm^−1^. ESI-MS (*m*/*z*) 329.1 (100, [M + Na]^+^), 307.2 (71, [M + H]^+^). HRMS (ESI-TOF) *m/z*: [M + H]^+^ calcd for C_19_H_19_N_2_O_2_ 307.1447, found 307.1445.

*3-Acetyl-4-(4′-nitrophenyl)-1-(p-tolyl)-pyrazole* (**15f**): light yellow solid, 144 mg (45%; for two steps, starting with 1.0 mmol of chalcone **10h** and chloride **14c**), mp 191–192 °C. ^1^H NMR (600 MHz, CDCl_3_) *δ* 2.44 (s, 3H, Me), 2.72 (s, 3H, Ac), 7.32–7.34, 7.65–7.67, 7.73–7.75 (3m, 2H each), 8.02 (s, 1H, 5-H), 8.23–8.25 (m, 2H). ^13^C NMR (151 MHz, CDCl_3_) *δ* 21.2, 27.9, 119.8, 123.5, 124.2, 128.4, 130.1, 130.4, 137.1, 138.4, 138.7, 147.2, 147.6, 194.7. IR (neat) *v* 1692, 1603, 1502, 1334, 1215, 1103, 1073 cm^−1^. ESI-MS (*m*/*z*) 344.9 (100, [M + Na]^+^). Anal. Calcd for C_18_H_15_N_3_O_3_ (321.1): C 67.28, H 4.71, N 13.08; found: C 67.35, H 4.93, N 12.95.

*3-Acetyl-1-(4′-methoxyphenyl)-4-phenylpyrazole* (**15g**): orange solid, 163 mg (56%; for two steps, starting with 1.0 mmol of chalcone **10a** and chloride **14d**), mp 109–111 °C. ^1^H NMR (600 MHz, CDCl_3_) *δ* 2.69 (s, 3H, Ac), 3.87 (s, 3H, OMe), 7.00–7.03, 7.32–7.35, 7.38–7.41, 7.55–7.57, 7.67–7.70 (5m, 2H, 1H, 2H, 2H, 2H), 7.88 (s, 1H, 5-H). ^13^C NMR (151 MHz, CDCl_3_) *δ* 28.0, 55.7, 114.8, 121.3, 126.3, 127.6, 128.0, 128.2, 129.3, 131.7, 133.3, 147.5, 159.2, 194.6. IR (neat) *v* 1685, 1513, 1466, 1353, 1260, 1221, 1174, 1118, 1029 cm^−1^. ESI-MS (*m*/*z*) 315.1 (92, [M + Na]^+^), 293.2 (100, [M + H]^+^). Anal. Calcd for C_18_H_16_N_2_O_2_ (292.1): C 73.95, H 5.52, N 9.58; found: C 73.99, H 5.74, N 9.49.

## 4. Conclusions

In summary, a solvent-free two-step mechanochemical synthesis of trifluoromethylated and non-fluorinated polysubstituted pyrazoles was developed, starting with simple substrates, i.e., chalcones and hydrazonoyl halides. The latter served as precursors for the K_2_CO_3_-induced in situ generation of nitrile imines, which were efficiently trapped with chalcones, to give the respective (3 + 2)-cycloadducts in moderate to high regioselectivity and fair yields. The first formed *trans*-configured 5-acylpyrazolines were oxidized with activated manganese dioxide under ball-milling to afford pyrazoles, formed through exclusive deacylative aromatization of the ring. Based on additional experiments, a mechanistic scenario comprising acyl-transfer onto the surface of heterogeneous oxidant was proposed. The presented results extend the scope of the previously reported method for the synthesis of the title compounds in organic solvents [42] and supplements recent developments, both in the synthesis of pyrazoles [2,53,54,55] and the application of nitrile imines as building blocks for organic synthesis [17,34,35,36,37,38,39,40,41,42,43,44,45,56,57,58,59].

## Figures and Tables

**Table 1:** Optimization of (3 + 2)-cycloaddition reaction of **9a** and **10a**.

Entry	Base	9a:10a:Base (Ratio)	Time (min)	V_jar_ (mL)	Conversion ^1^ (%)	Ratio (%) ^1^ (Isolated Yield)	
						6a	6′a
1	Et_3_N	1.1:1.0:1.2	60	1.5	56 ^2^	87	13
2	CsF	1.1:1.0:1.2	60	1.5	27 ^2^	72	28
3	KF	1.1:1.0:1.2	60	1.5	60 ^2^	84	16
4	Cs_2_CO_3_	1.1:1.0:1.2	60	1.5	45 ^2^	79	21
5	K_2_CO_3_	1.1:1.0:1.2	60	1.5	82	81	19
6	K_2_CO_3_	1.1:1.0:1.2	90	1.5	84	82	18
7	K_2_CO_3_	1.2:1.0:1.3	90	5	93	80 (73)	20 (13)
8	K_2_CO_3_	1.2:1.0:1.3	180	5	93	82 (75)	18 (13)

^1^ Estimated based on ^1^H NMR spectra of crude reaction mixtures; ^2^ Partial decomposition of starting bromide **9a**.

**Table 2:** Ball-milling (3 + 2)-cycloadditions of **9b**–**9h** with model chalcone (**10a**).

Entry	Substrate	X	6:6′ Ratio ^1^	Yield of 6 (%) ^2^
1	**9b**	4-Me	81:19	**6b** (70)
2	**9c**	4-*i*-Pr	81:19	**6c** (71)
3	**9d**	4-OBn	83:17	**6d** (58)
4	**9e**	4-Cl	81:19	**6e** (53)
5	**9f**	2,4-Cl_2_	77:23	**6f** (54)
6	**9g**	4-NO_2_	74:26	**6g** (10)
7 ^3^	**9g**	4-NO_2_	79:21	**6g** (22)
8 ^3^	**9h**	4-PhCOO	79:21	**6h** (17)

^1^ Estimated on the basis of ^1^H NMR spectra of crude reaction mixtures; ^2^ Isolated yield; ^3^ Grinding time 24 h.

**Table 3:** Mechanochemical (3 + 2)-cycloadditions of **10b**–**10o** with model nitrile imine **1b**.

Entry	Substrate	R	R’	Milling Time (h)	6:6′ Ratio (%) ^1^	Yield of 6 (%) ^2^
1	**10b**	2-Nph ^3^	Ph	9	77:23	**6i** (74)
2	**10c**	Fc ^3^	Ph	24	85:15	**6j** (38)
3	**10d**	4-MeOC_6_H_4_	Ph	20	76:24	**6k** (59)
4	**10e**	3,4-(MeO)_2_C_6_H_3_	Ph	18	100:0	**6l** (74)
5	**10f**	3,4-methylenedioxyphenyl	Ph	12	88:12	**6m** (68)
6	**10g**	4-(Me_2_N)C_6_H_4_	Ph	36	65:35	**6n** (46)
7	**10h**	4-ClC_6_H_4_	Ph	9	79:21	**6o** (70)
8	**10i**	2-ClC_6_H_4_	Ph	10	77:23	**6p** (57)
9	**10j**	4-CF_3_C_6_H_4_	Ph	9	71:29	**6q** (28)
10	**10k**	4-NO_2_C_6_H_4_	Ph	28	73:27	**6r** (26)
11	**10l**	3-NO_2_C_6_H_4_	Ph	72	79:21	**6s** (65)
12	**10m**	Ph	Fc ^3^	24	71:29	**6t** (39)
13	**10n**	Ph	4-BrC_6_H_4_	16	85:15	**6u** (68)
14	**10o**	Ph	3,4-methylenedioxyphenyl	20	82:18	**6v** (81)

^1^ Estimated based on ^1^H NMR spectra of crude reaction mixtures; ^2^ Isolated yield; ^3^ 2-Nph = naphth-2-yl; Fc = ferrocenyl.

## Data Availability

All the electronic experimental data and samples of new materials are available from the authors.

## Supplementary Materials

The following supporting information can be downloaded at: https://www.mdpi.com/article/10.3390/molecules27238446/s1: Copies of ^1^H, ^13^C, and ^19^F NMR spectra of all new compounds.
