# Supplementary material for: Fluorinated and Non-Fluorinated 1,4-Diarylpyrazoles via MnO2-Mediated Mechanochemical Deacylative Oxidation of 5-Acylpyrazolines
Source: Molecules. 2022 Dec 2;27(23):8446. doi: 10.3390/molecules27238446 (PMC9736116; doi:10.3390/molecules27238446)
Supplement: Supplementary file 1 [file molecules-27-08446-s001.zip › molecules-2045781-supplementary.pdf]

**Supporting Information**

for

# Fluorinated and non-fluorinated 1,4-diarylpyrazoles via $MnO_2$-mediated mechanochemical deacylative oxidation of 5-acylpyrazolines

Greta Utecht-Jarzyńska,[a] Anna Kowalczyk,[a,b] Marcin Jasiński[a,*]

[a] *Faculty of Chemistry, University of Lodz, Tamka 12, 91403 Łódź, Poland*

[b] *The University of Lodz Doctoral School of Exact and Natural Sciences, Banacha 12/16, 90237 Łódź, Poland*

[*] Corresponding author: Marcin Jasiński – *University of Lodz, Faculty of Chemistry, Łódź, Poland;*

https://orcid.org/0000-0002-8789-9690;

Phone: +48-42-635 5766;

Email: mjasinski@uni.lodz.pl

**Content**

Copies of $^{1}H$ and $^{13}C$ NMR spectra of new compounds S2

Copies of $^{19}F$ NMR spectra of new pyrazolines and pyrazoles S22

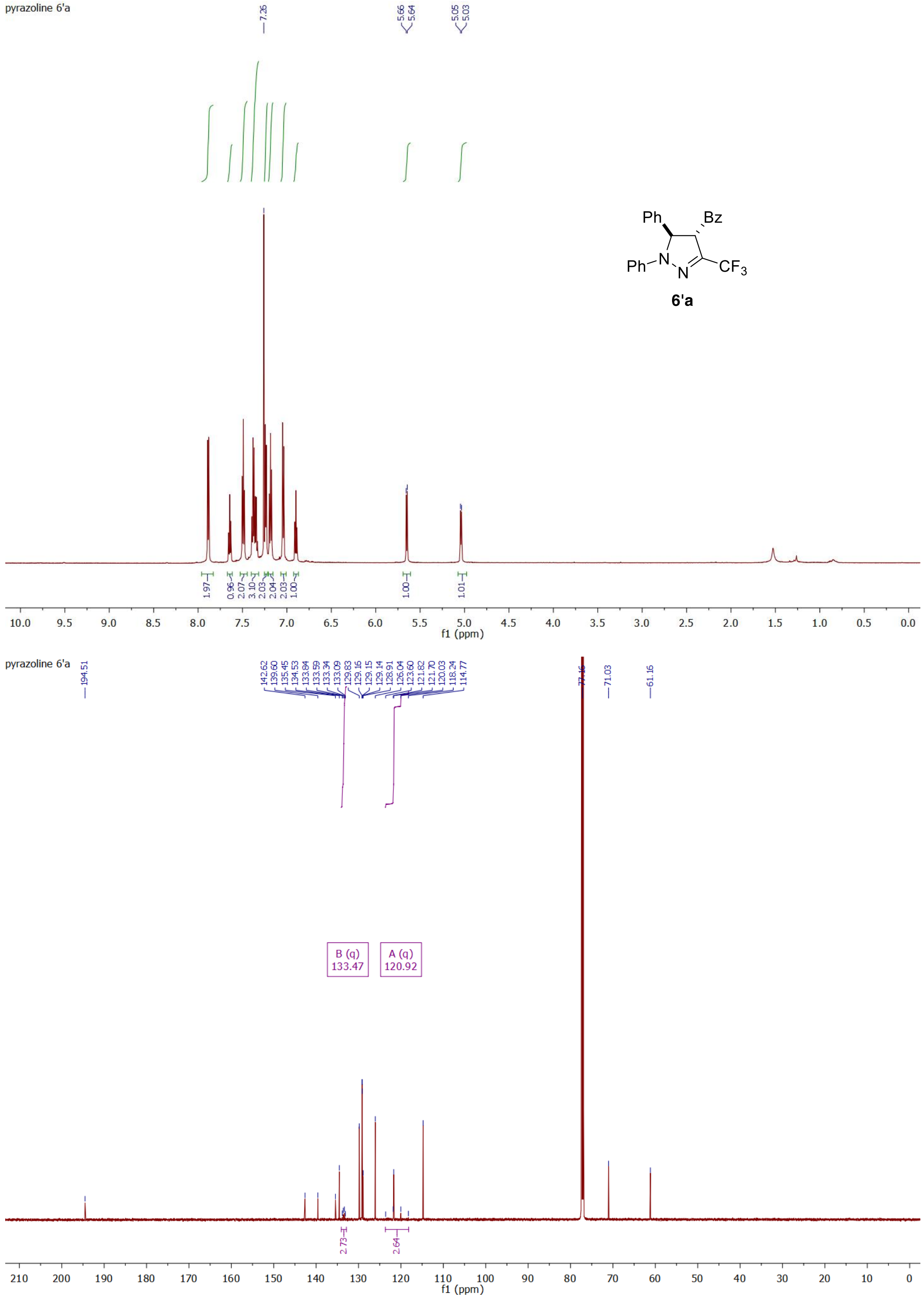


**Figure S1**. $^{1}H$ NMR (600 MHz, $CDCl_3$) and $^{13}C$ NMR (151 MHz, $CDCl_3$) spectra for compound **6’a**.

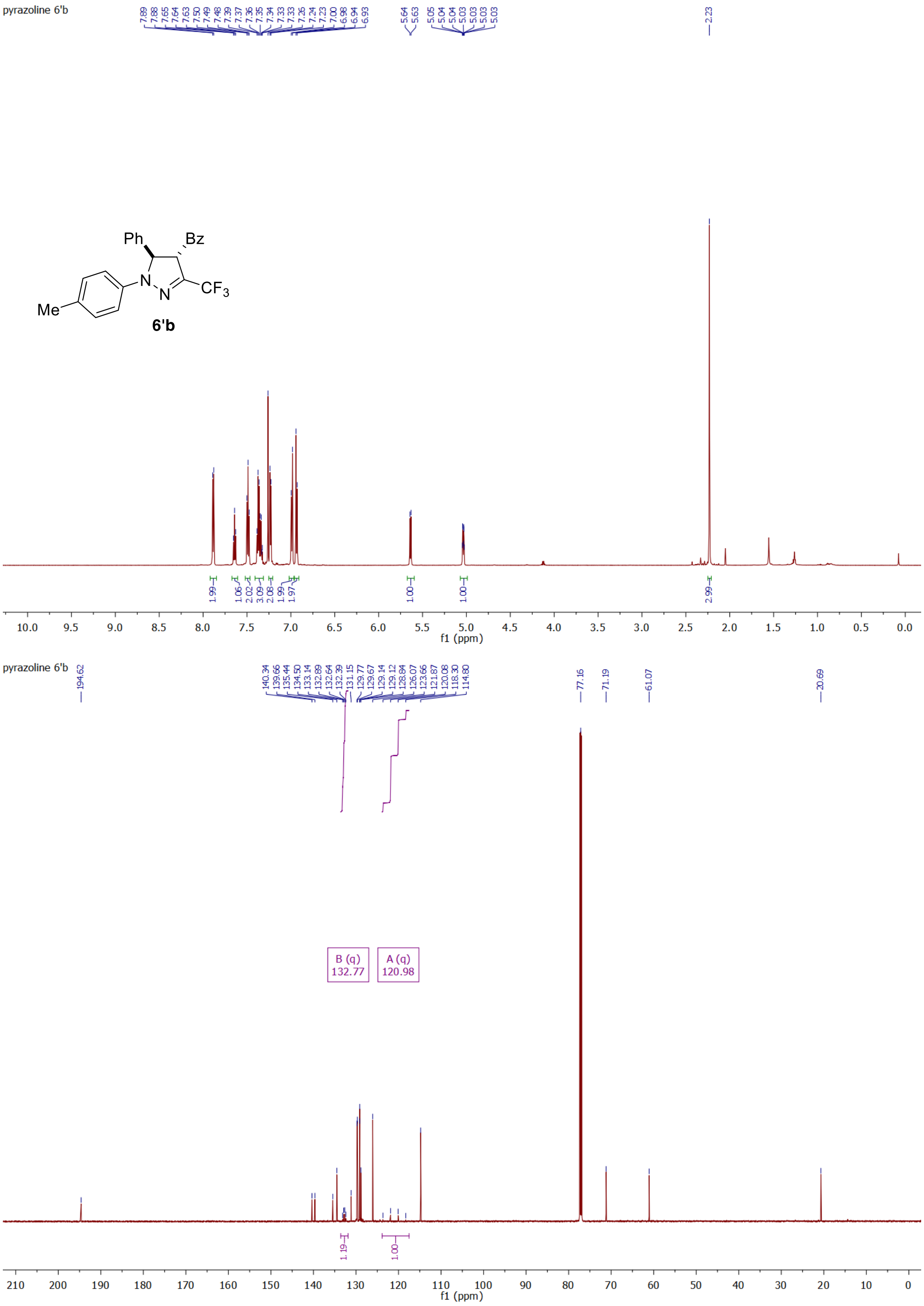


**Figure S2**. $^{1}H$ NMR (600 MHz, $CDCl_3$) and $^{13}C$ NMR (151 MHz, $CDCl_3$) spectra for compound **6'b**.

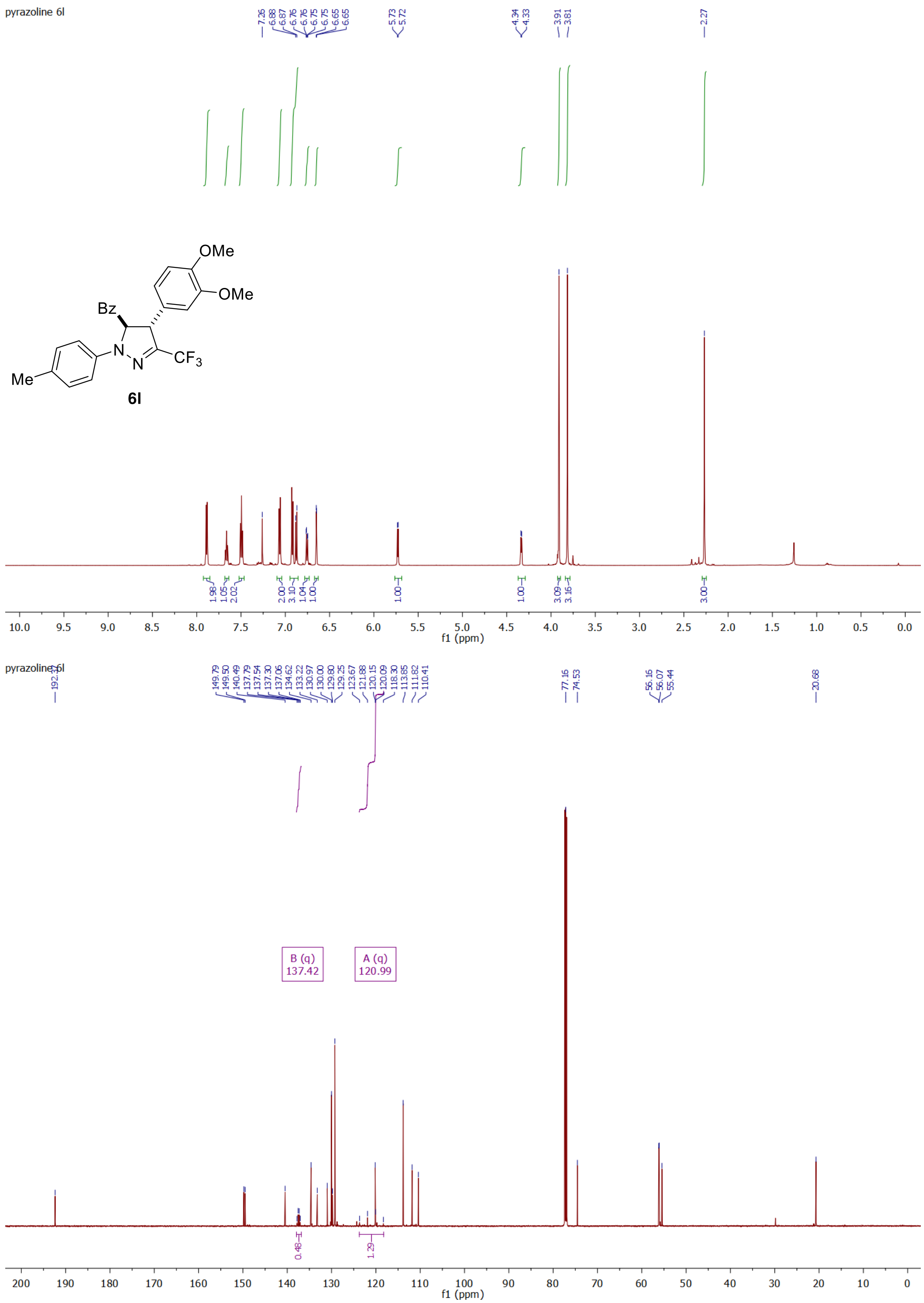


**Figure S3**. $^{1}H$ NMR (600 MHz, $CDCl_3$) and $^{13}C$ NMR (151 MHz, $CDCl_3$) spectra for compound **6l**.

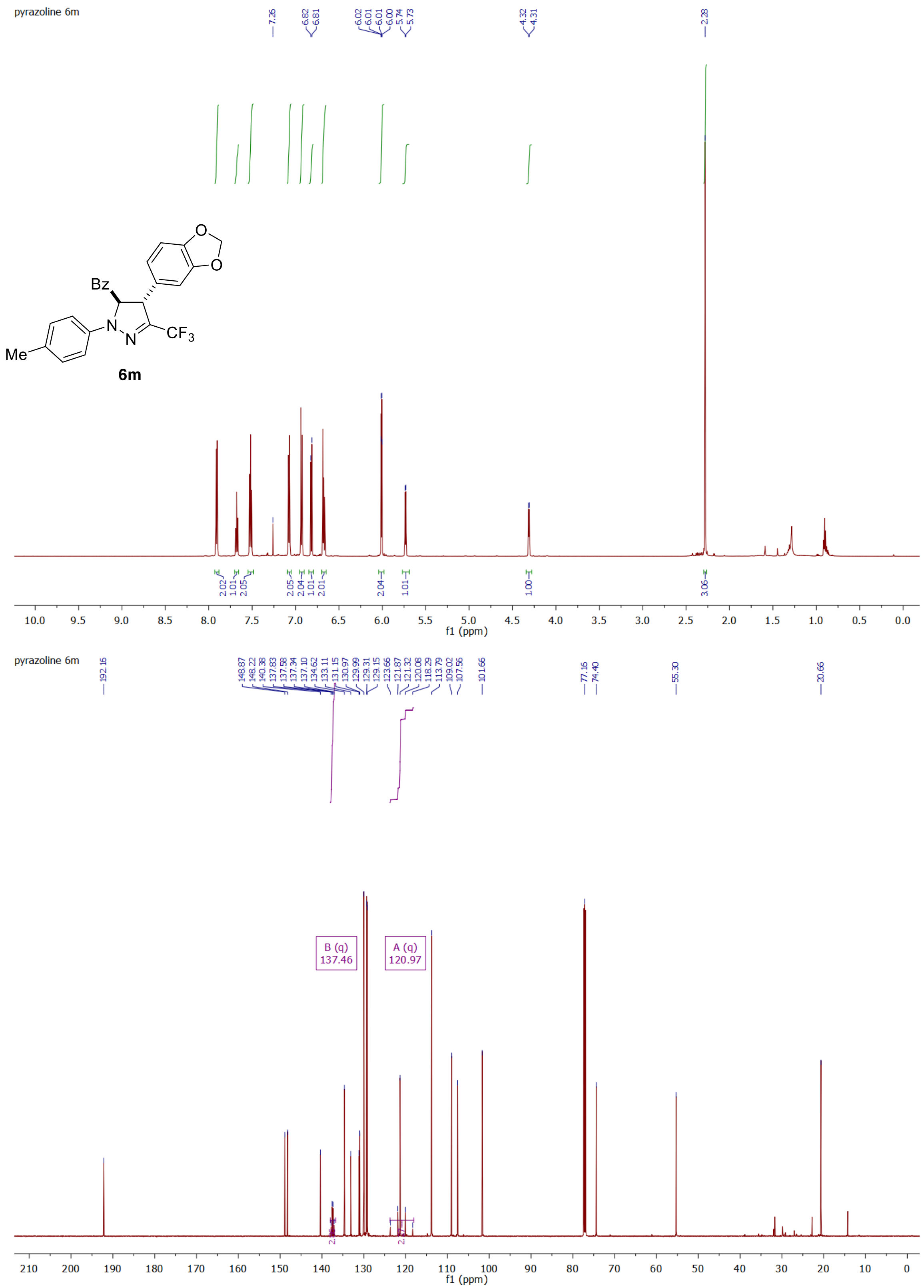


**Figure S4**. $^{1}$H NMR (600 MHz, $CDCl_3$) and $^{13}$C NMR (151 MHz, $CDCl_3$) spectra for compound **6m**.

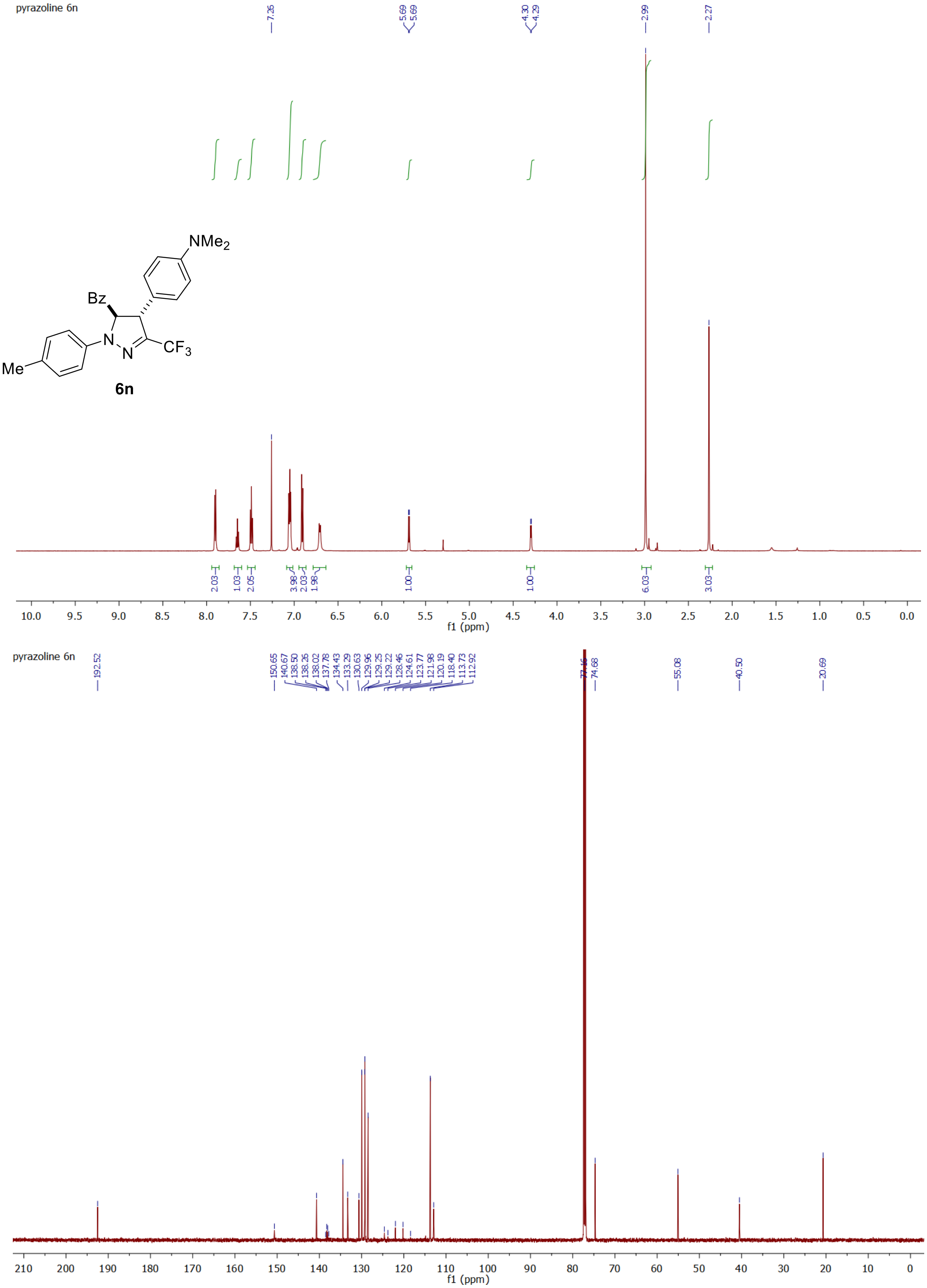


**Figure S5**. $^{1}H$ NMR (600 MHz, $CDCl_3$) and $^{13}C$ NMR (151 MHz, $CDCl_3$) spectra for compound **6n**.

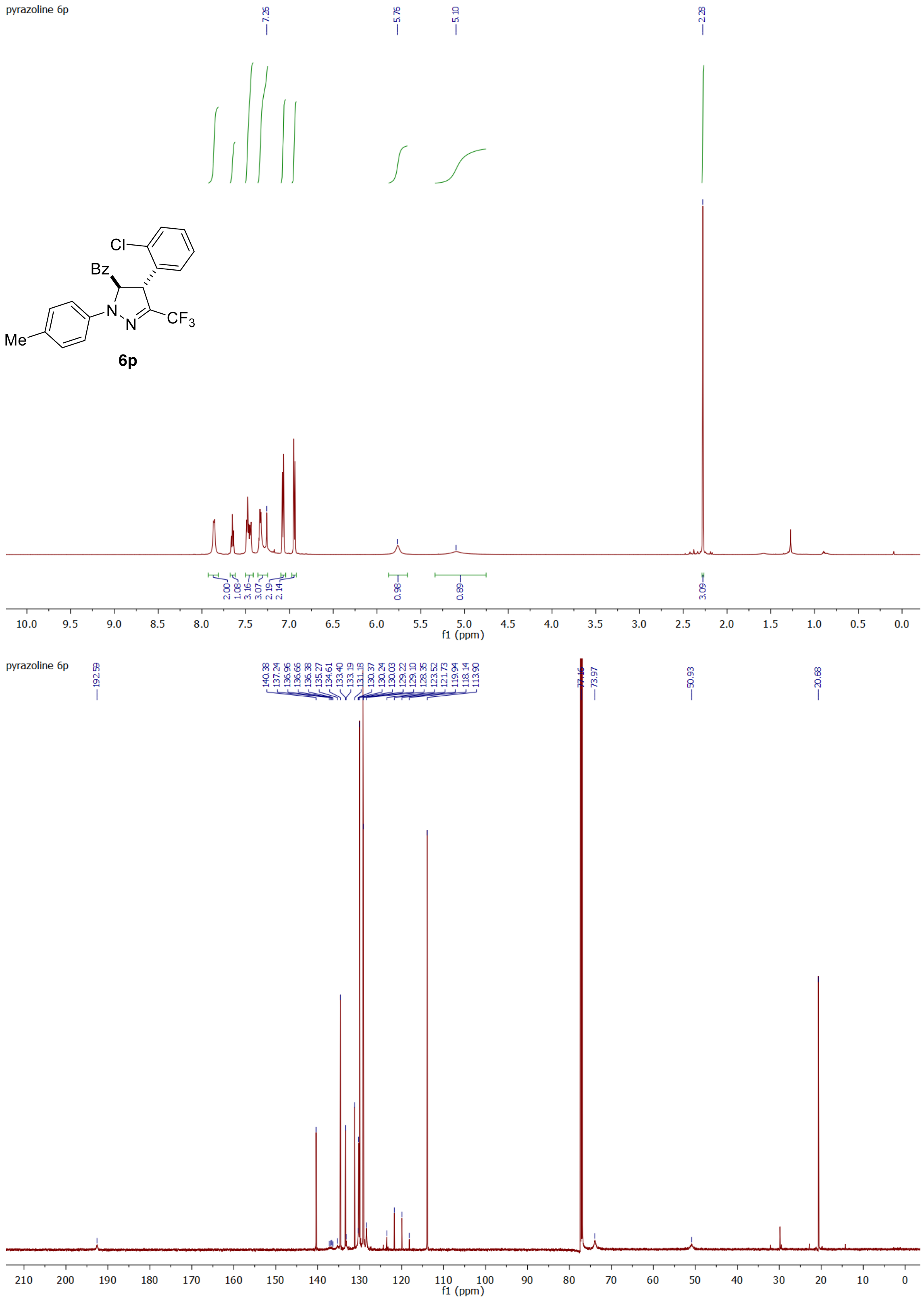


**Figure S6**. $^{1}H$ NMR (600 MHz, $CDCl_3$) and $^{13}C$ NMR (151 MHz, $CDCl_3$) spectra for compound **6p**.

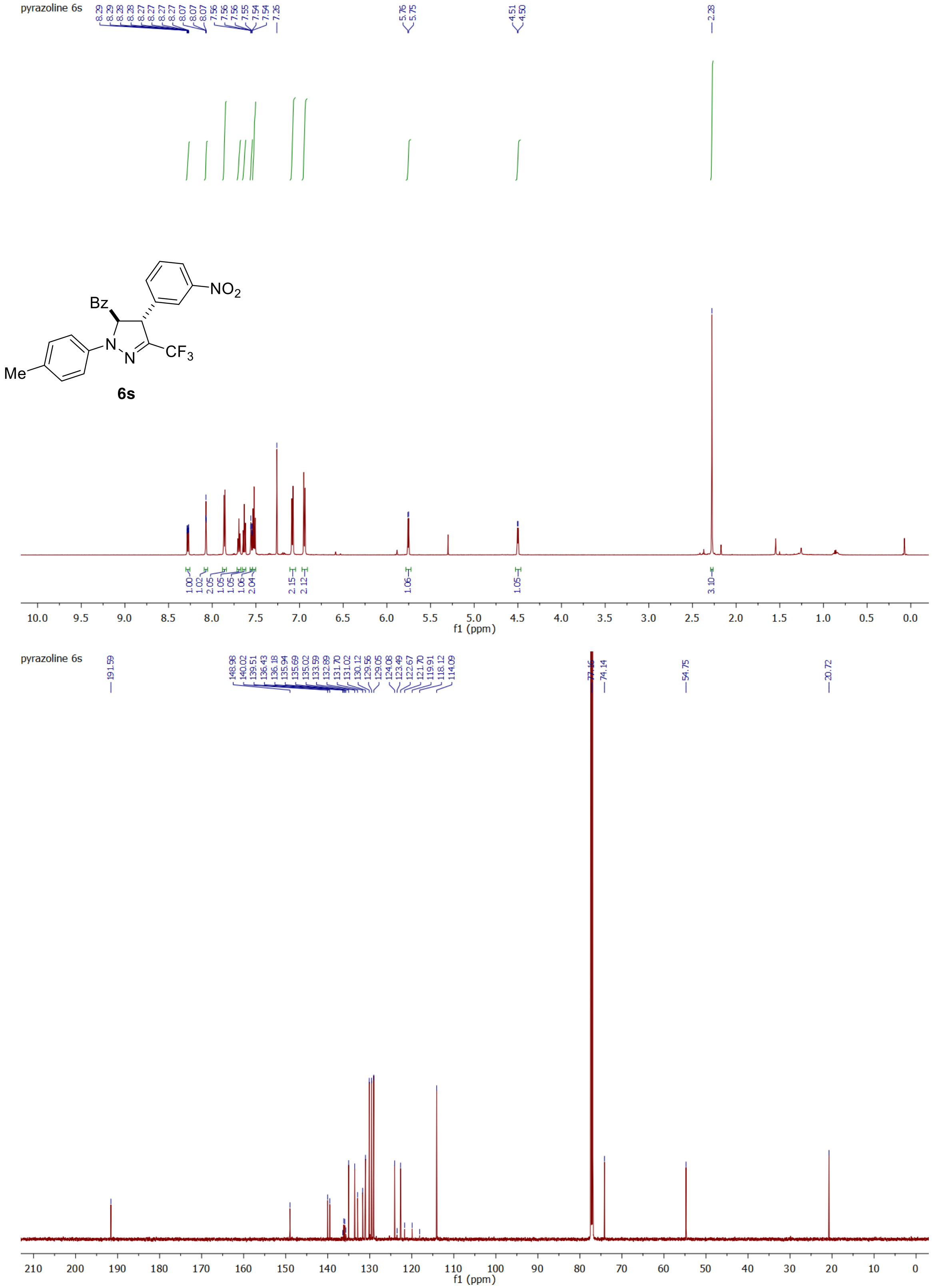


**Figure S7**. $^{1}$H NMR (600 MHz, $CDCl_3$) and $^{13}$C NMR (151 MHz, $CDCl_3$) spectra for compound **6s**.

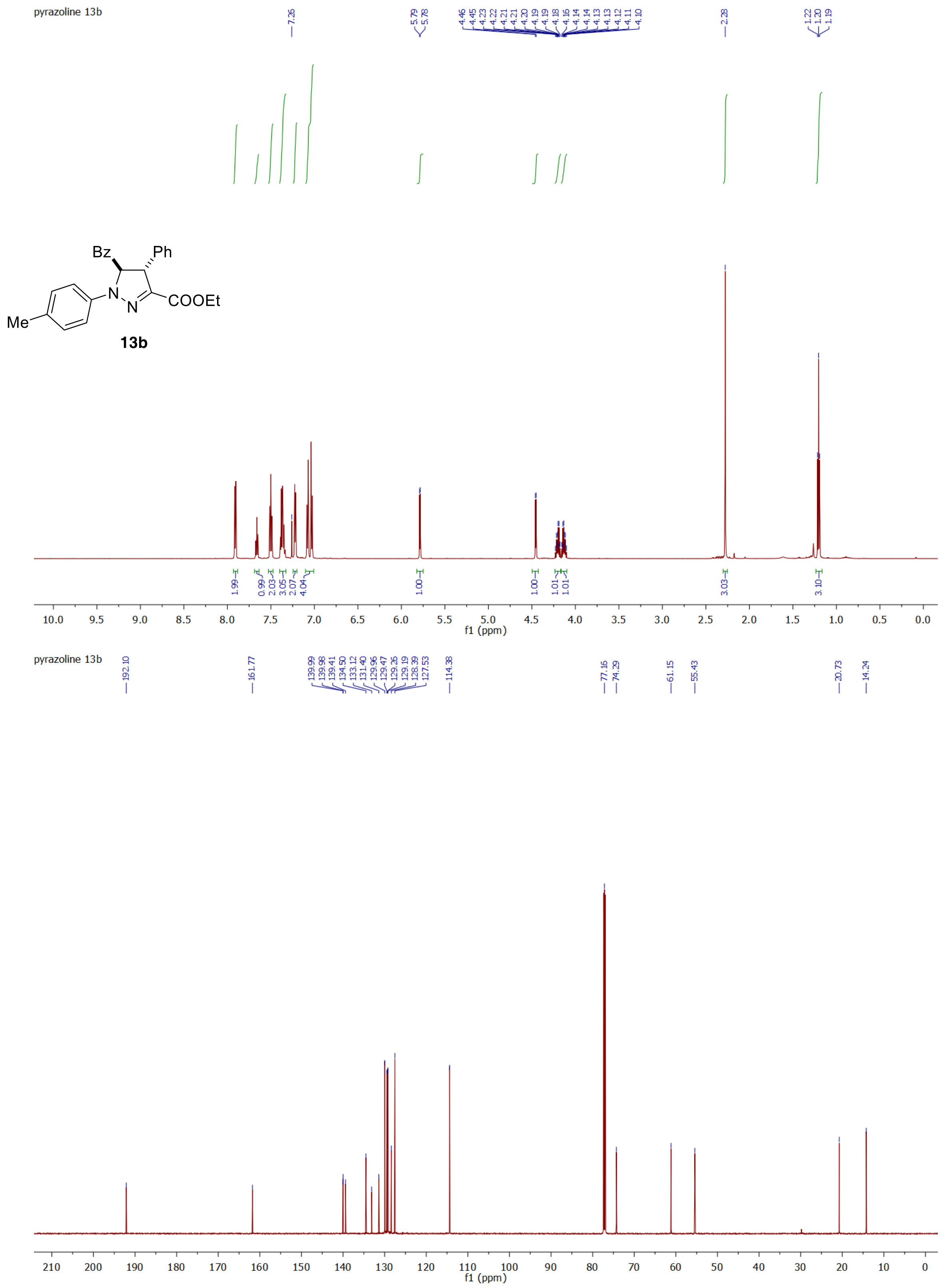


**Figure S8**. $^{1}H$ NMR (600 MHz, $CDCl_3$) and $^{13}C$ NMR (151 MHz, $CDCl_3$) spectra for compound **13b**.

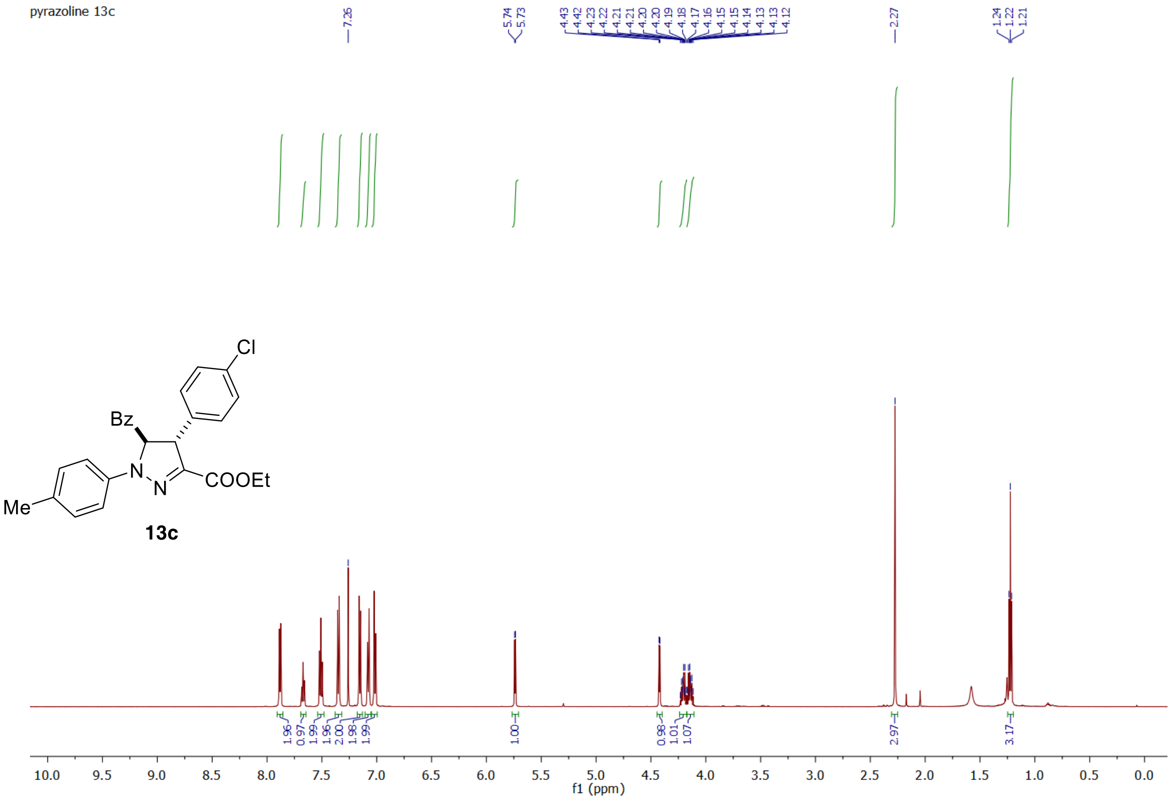


13c

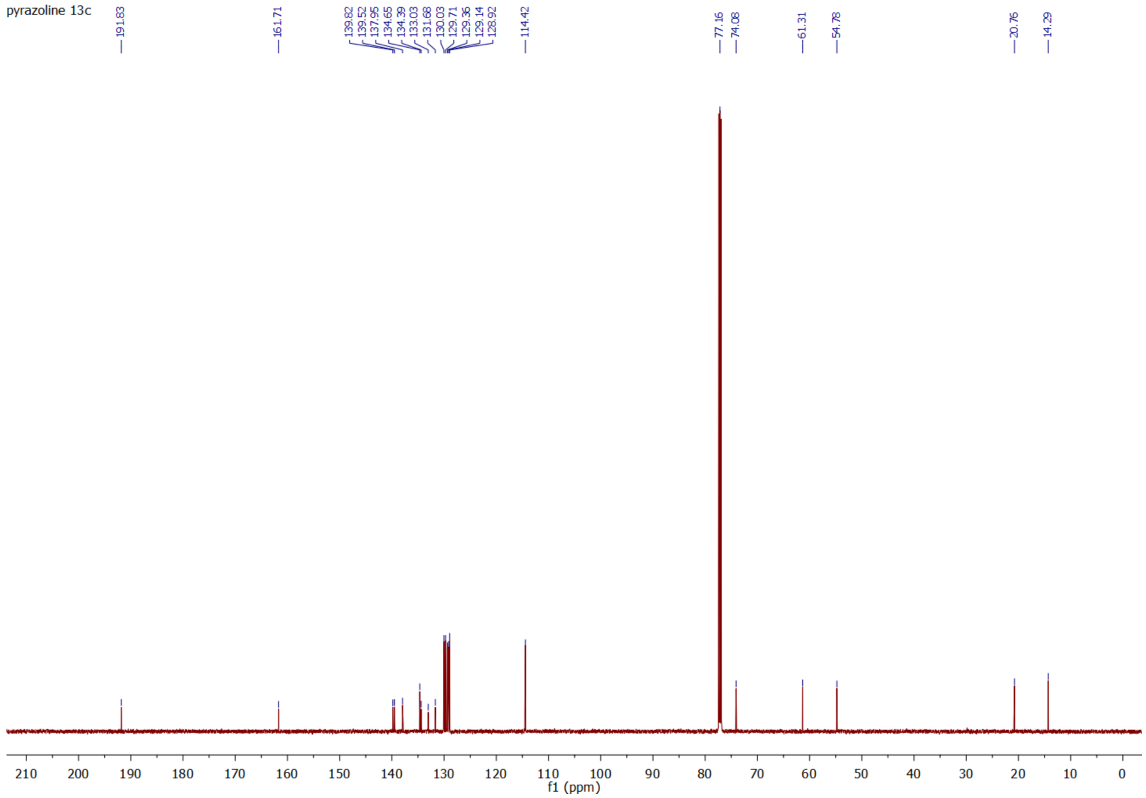


**Figure S9**. $^{1}$H NMR (600 MHz, $CDCl_3$) and $^{13}$C NMR (151 MHz, $CDCl_3$) spectra for compound **13c**.

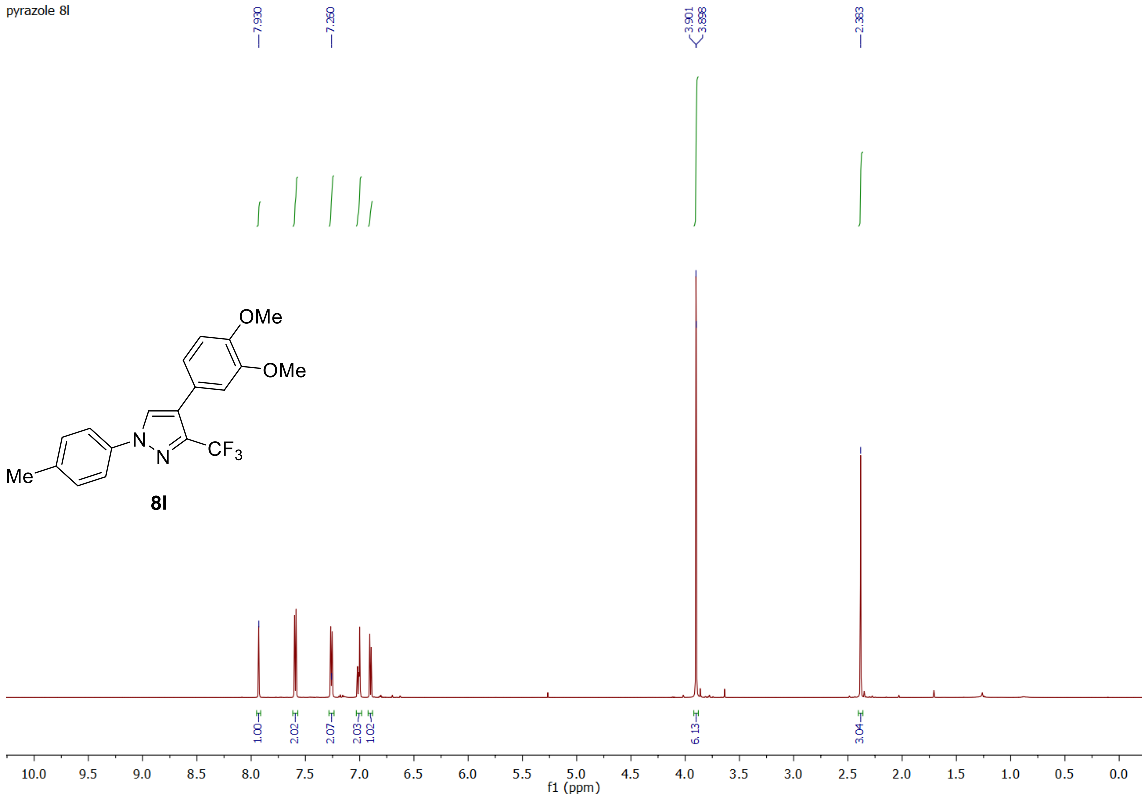


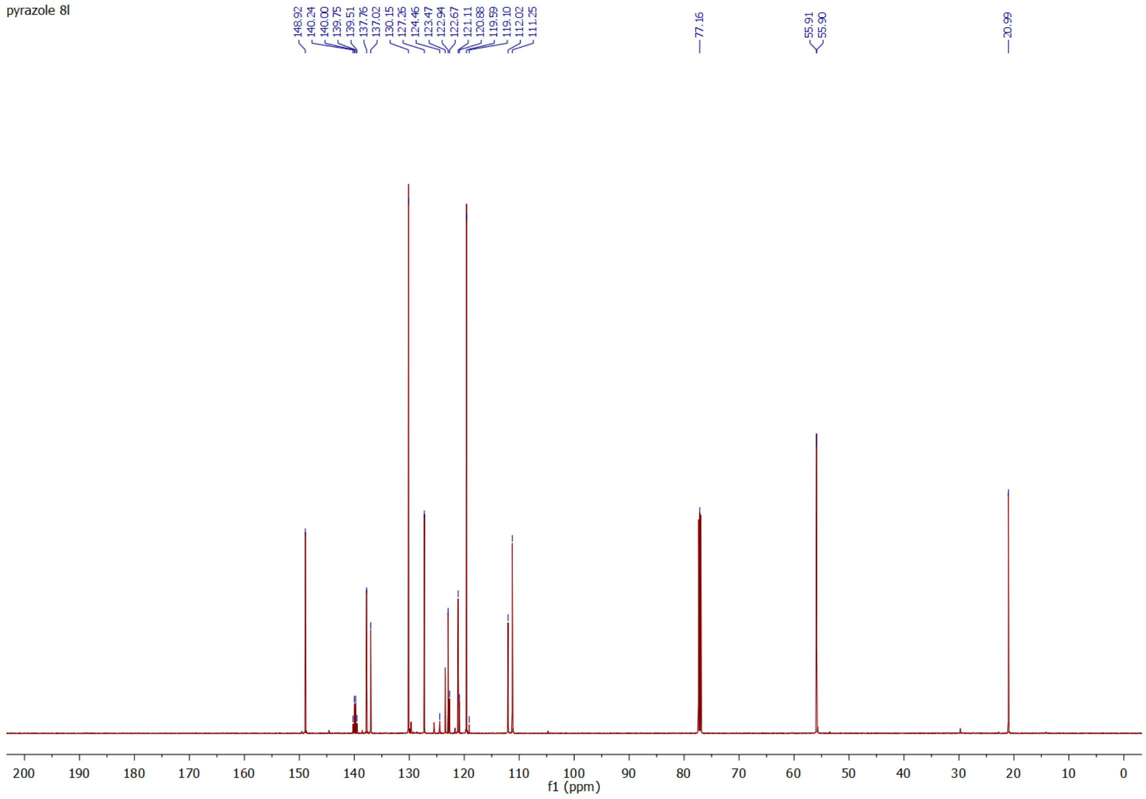


**Figure S10**. $^{1}H$ NMR (600 MHz, $CDCl_3$) and $^{13}C$ NMR (151 MHz, $CDCl_3$) spectra for compound **8l**.

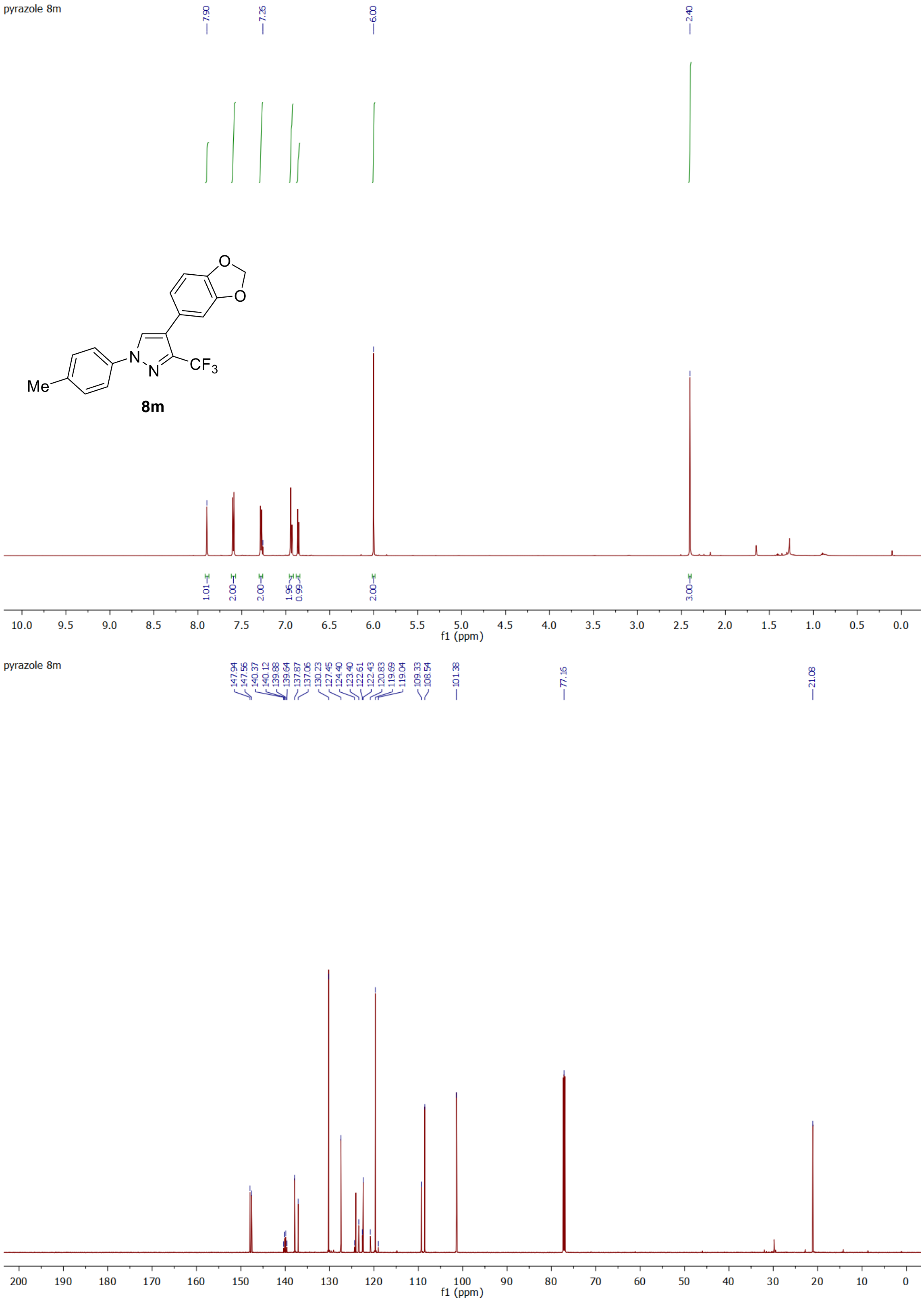


**Figure S11**. $^{1}H$ NMR (600 MHz, $CDCl_3$) and $^{13}C$ NMR (151 MHz, $CDCl_3$) spectra for compound **8m**.

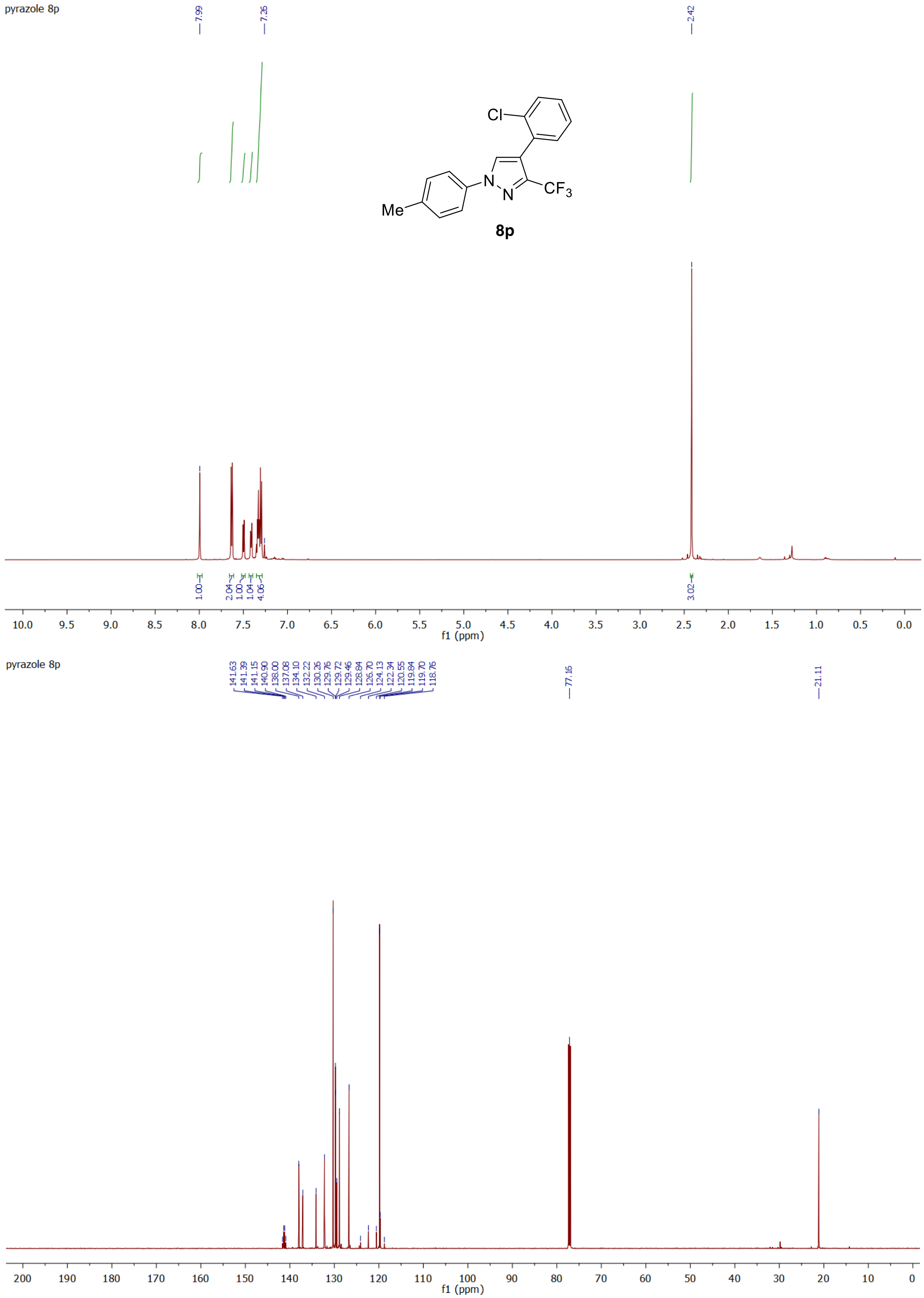


**Figure S12**. $^{1}H$ NMR (600 MHz, $CDCl_3$) and $^{13}C$ NMR (151 MHz, $CDCl_3$) spectra for compound **8p**.

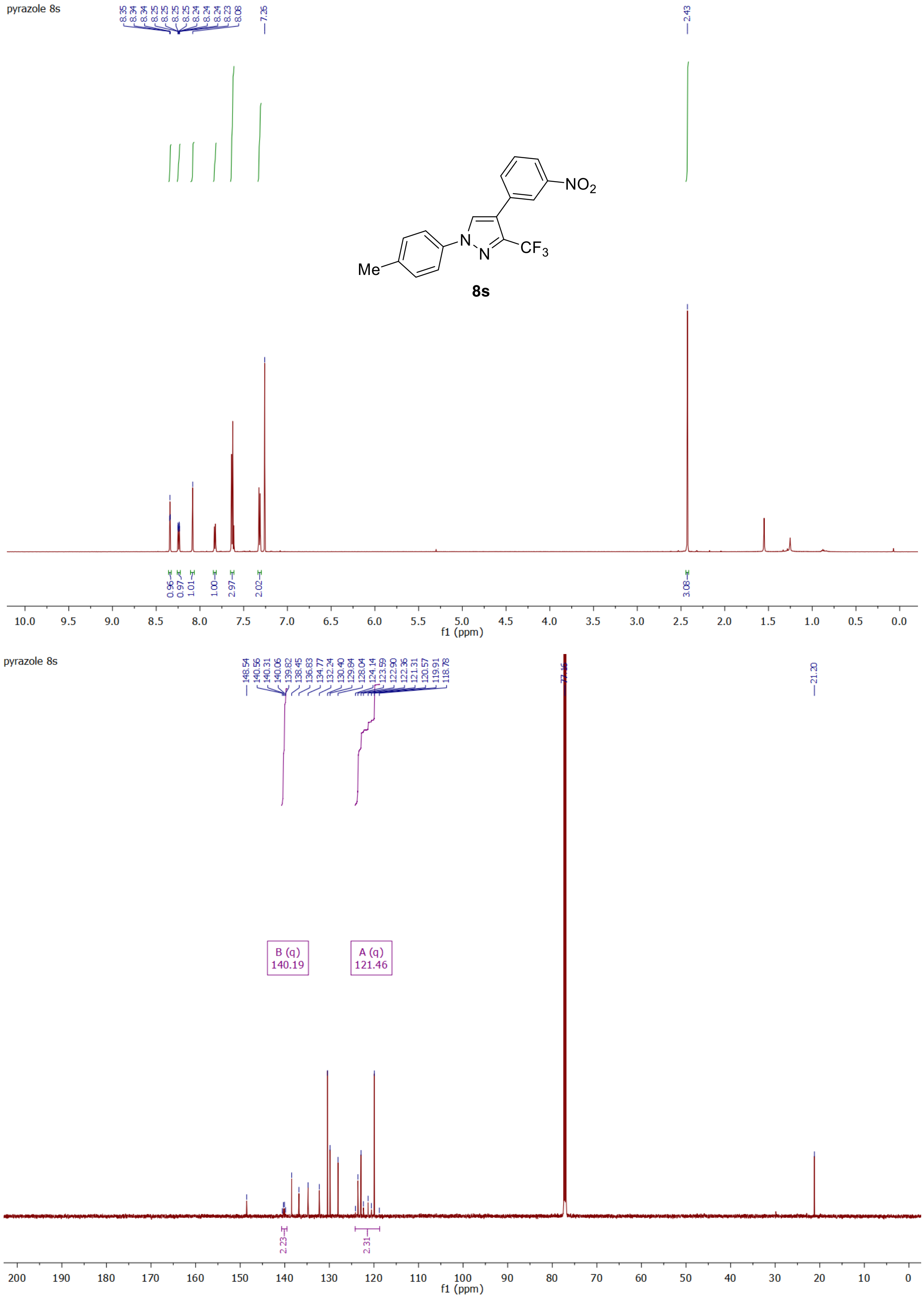


**Figure S13**. $^{1}H$ NMR (600 MHz, $CDCl_3$) and $^{13}C$ NMR (151 MHz, $CDCl_3$) spectra for compound **8s**.

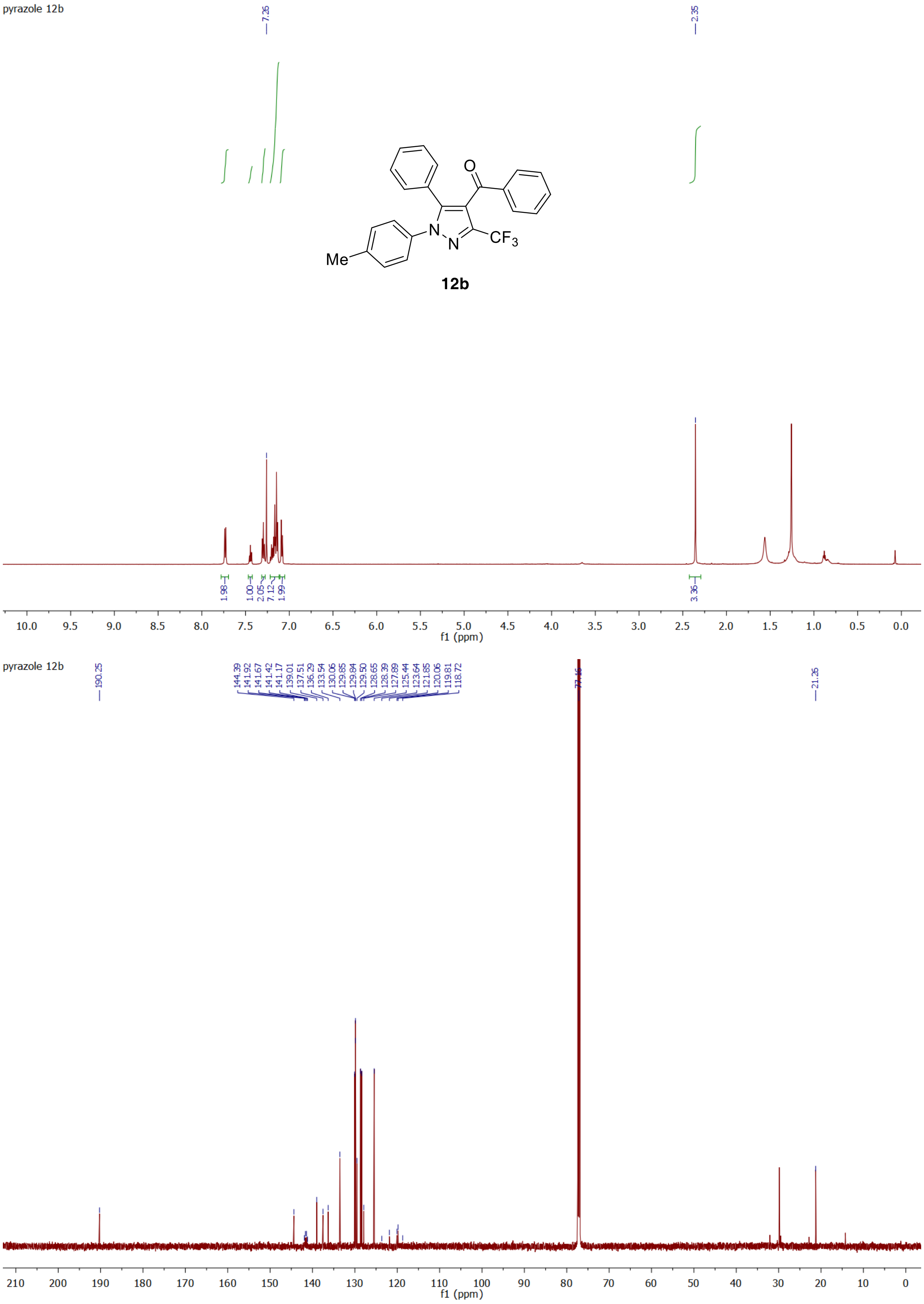


**Figure S14**. $^{1}H$ NMR (600 MHz, $CDCl_3$) and $^{13}C$ NMR (151 MHz, $CDCl_3$) spectra for compound **12b**.

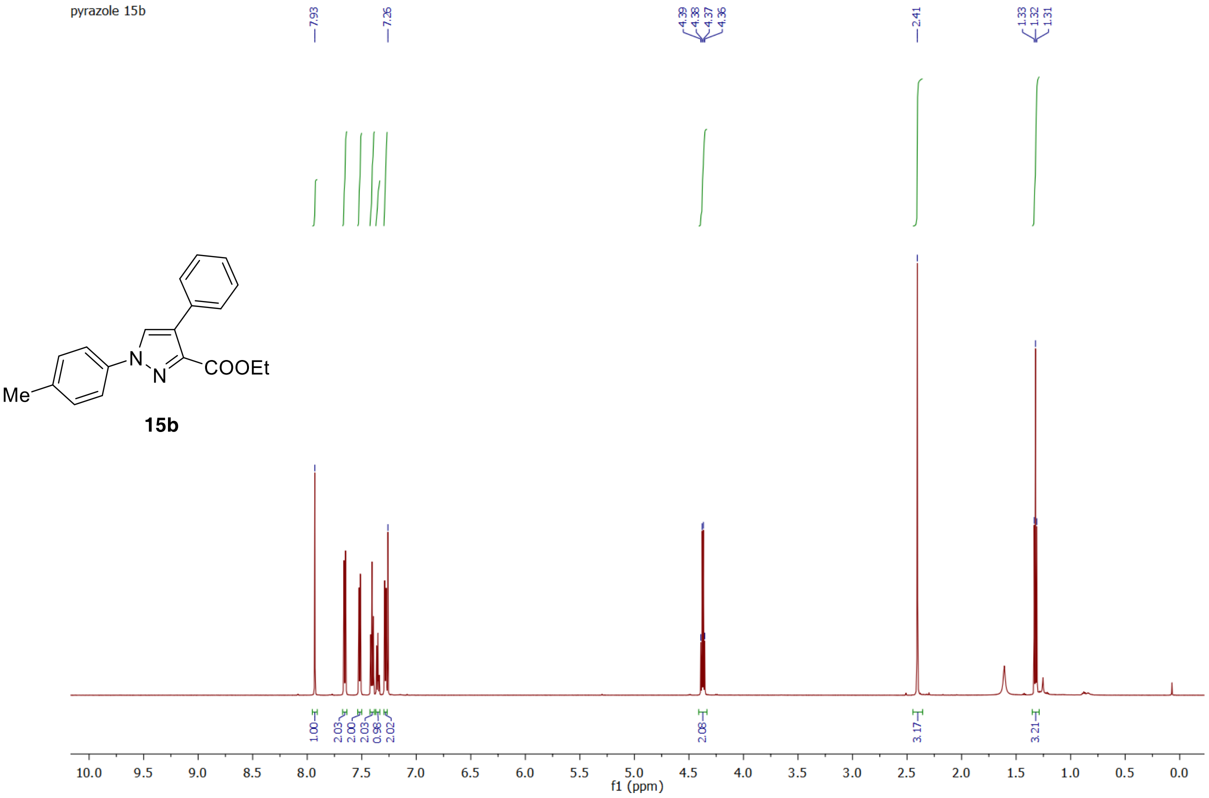


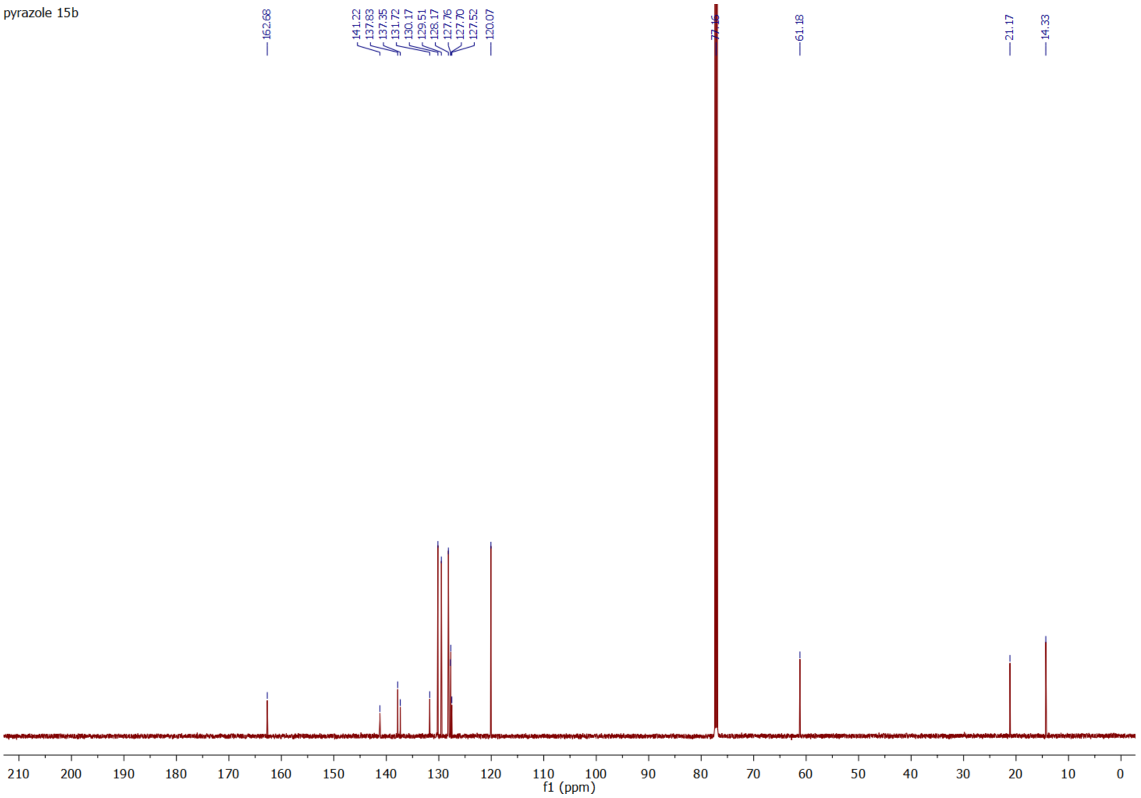


**Figure S15**. $^{1}H$ NMR (600 MHz, $CDCl_3$) and $^{13}C$ NMR (151 MHz, $CDCl_3$) spectra for compound **15b**.

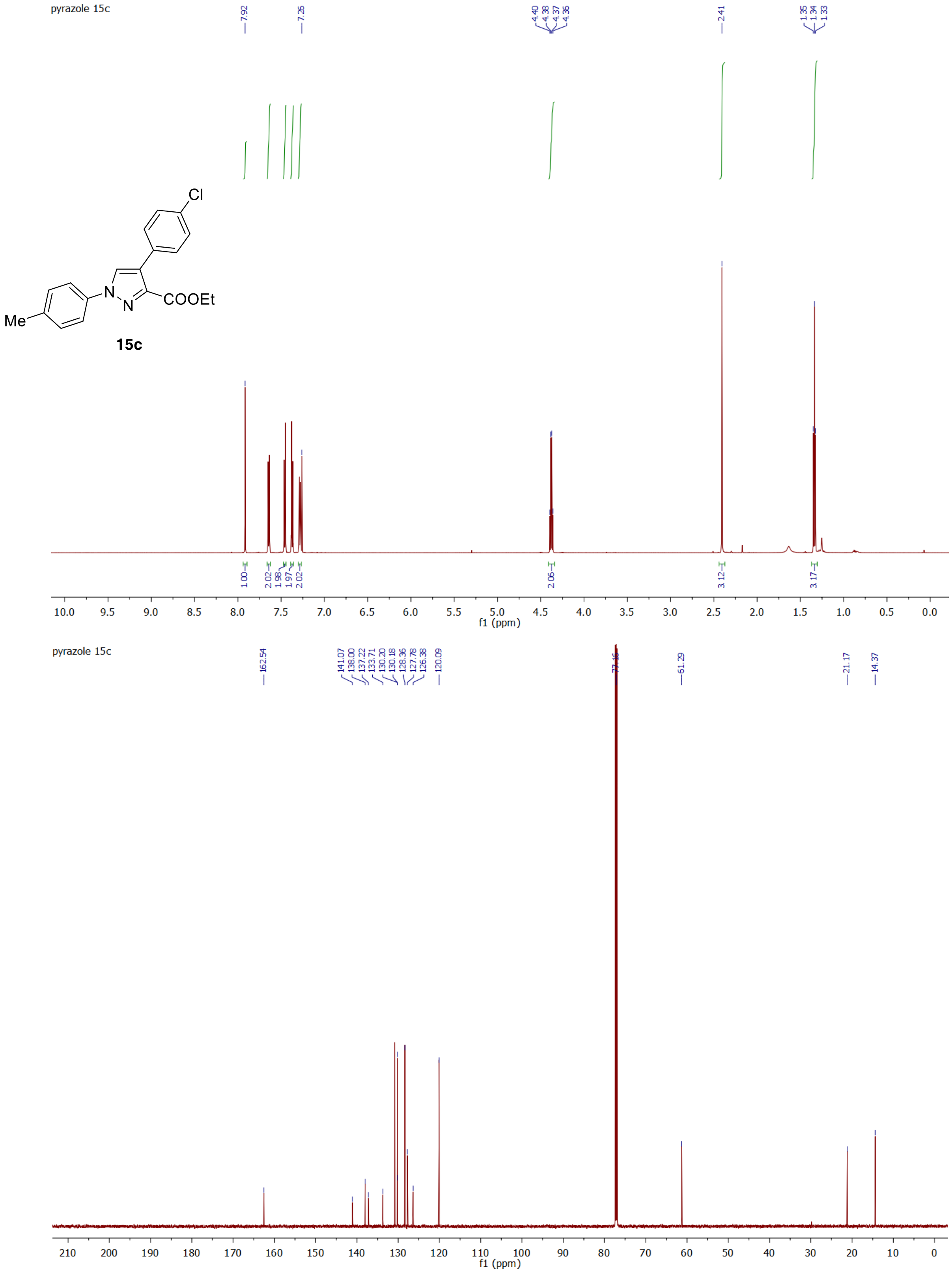


**Figure S16**. $^{1}H$ NMR (600 MHz, $CDCl_3$) and $^{13}C$ NMR (151 MHz, $CDCl_3$) spectra for compound **15c**.

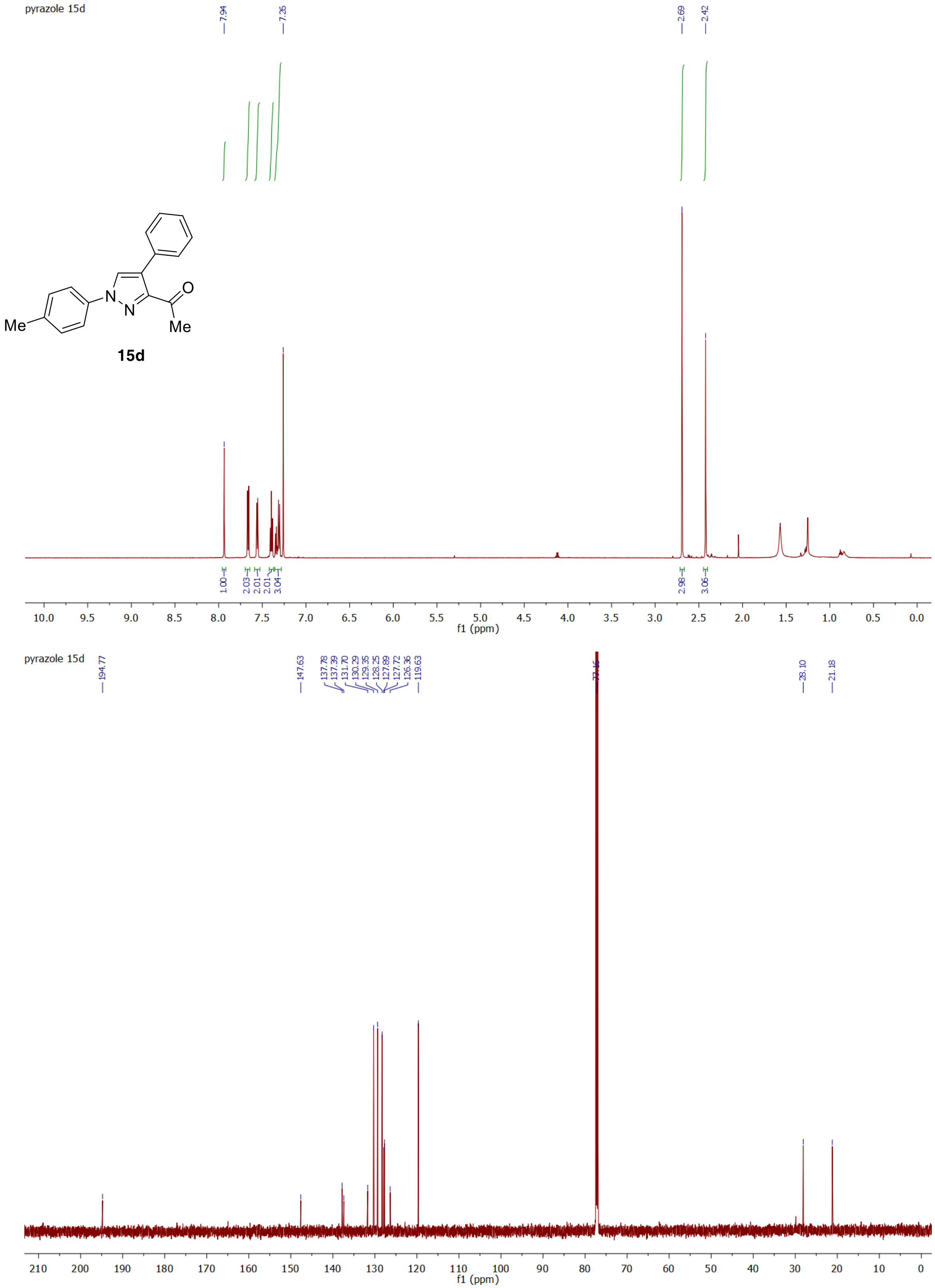


**Figure S17**. $^{1}H$ NMR (600 MHz, $CDCl_3$) and $^{13}C$ NMR (151 MHz, $CDCl_3$) spectra for compound **15d**.

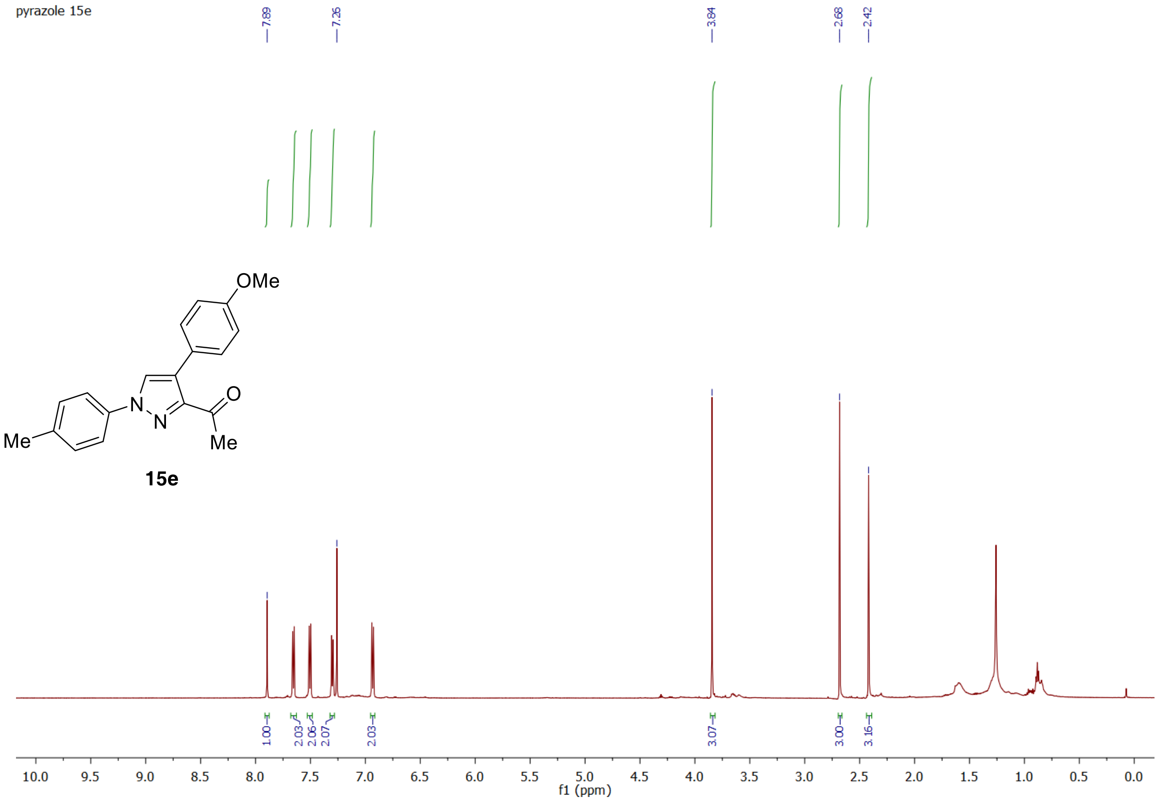


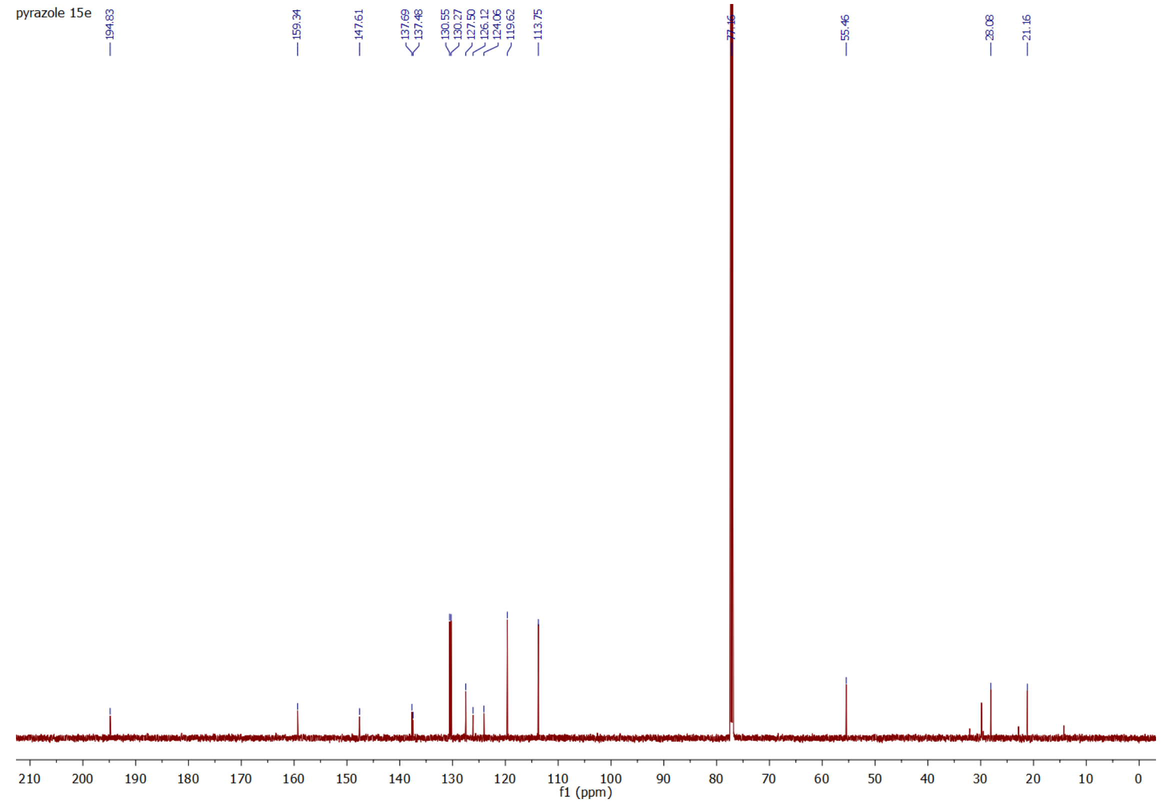


**Figure S18**. $^{1}$H NMR (600 MHz, $CDCl_3$) and $^{13}$C NMR (151 MHz, $CDCl_3$) spectra for compound **15e**.

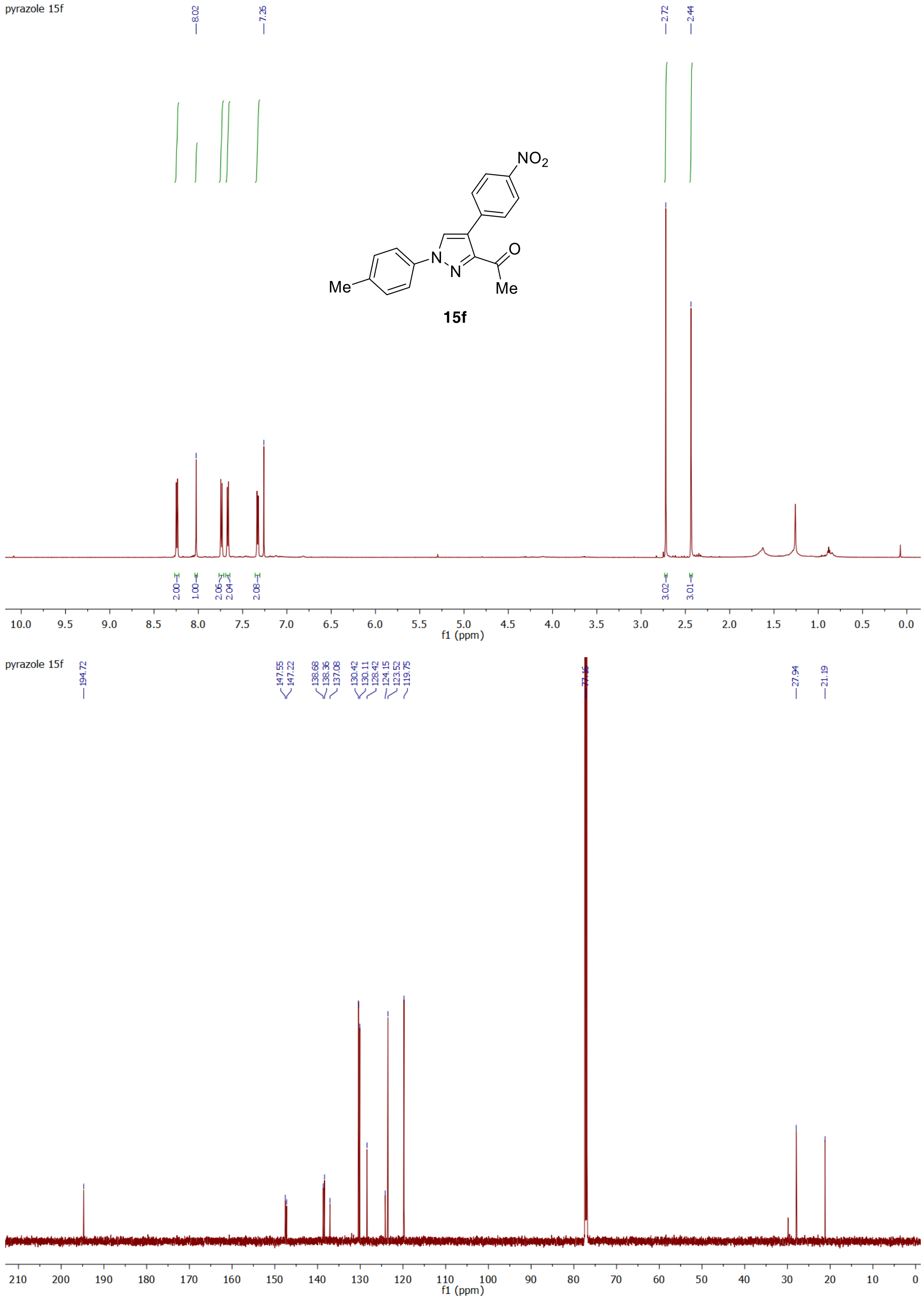


**Figure S19**. ¹H NMR (600 MHz, $CDCl_3$) and ¹³C NMR (151 MHz, $CDCl_3$) spectra for compound **15f**.

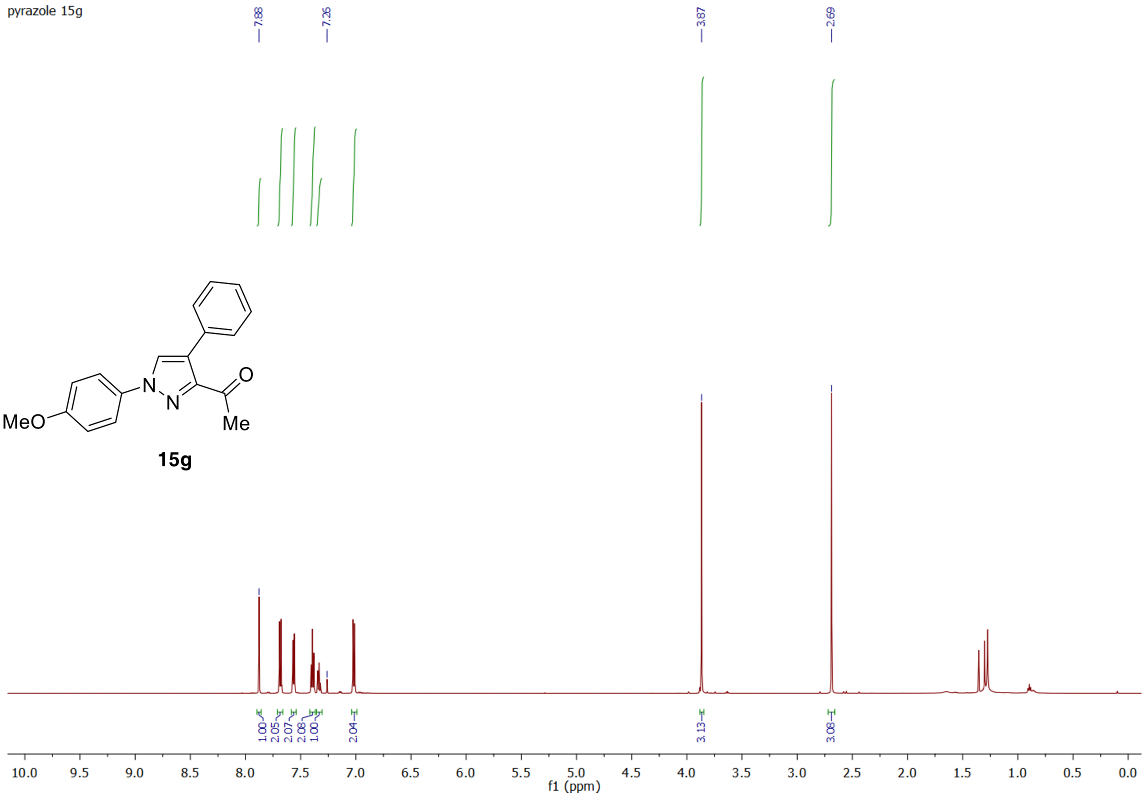


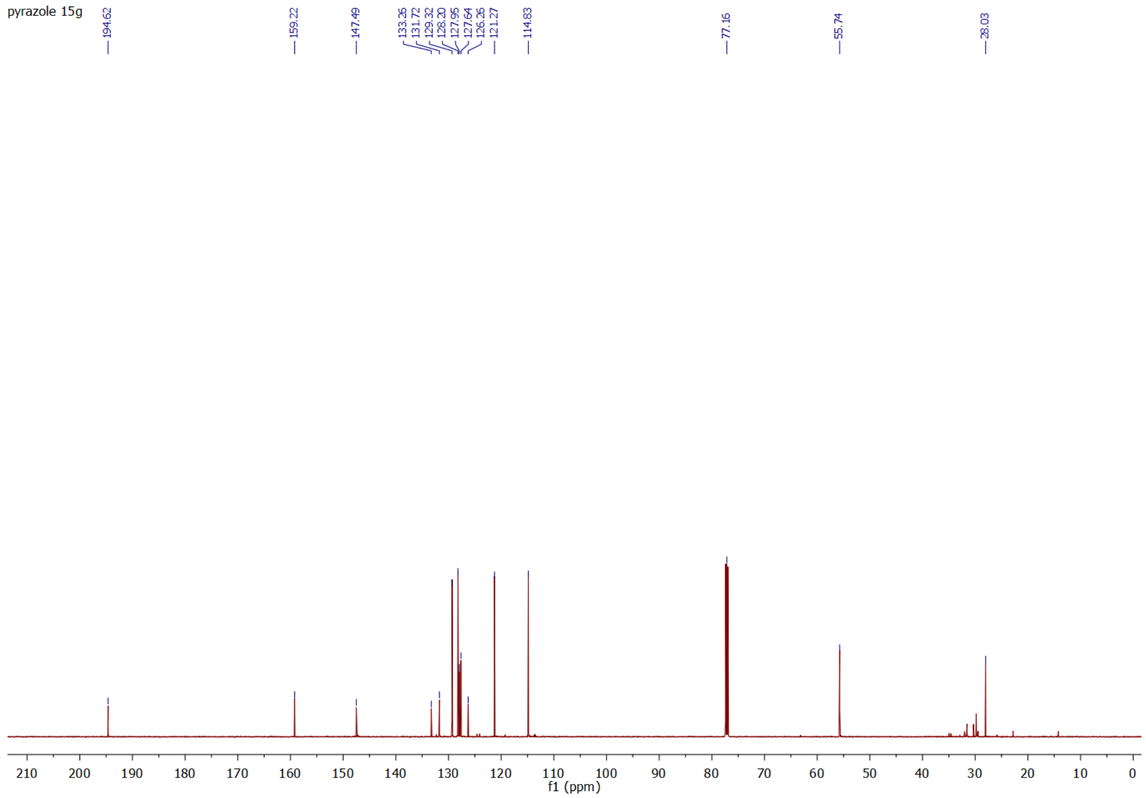


**Figure S20**. $^{1}H$ NMR (600 MHz, $CDCl_3$) and $^{13}C$ NMR (151 MHz, $CDCl_3$) spectra for compound **15g**.

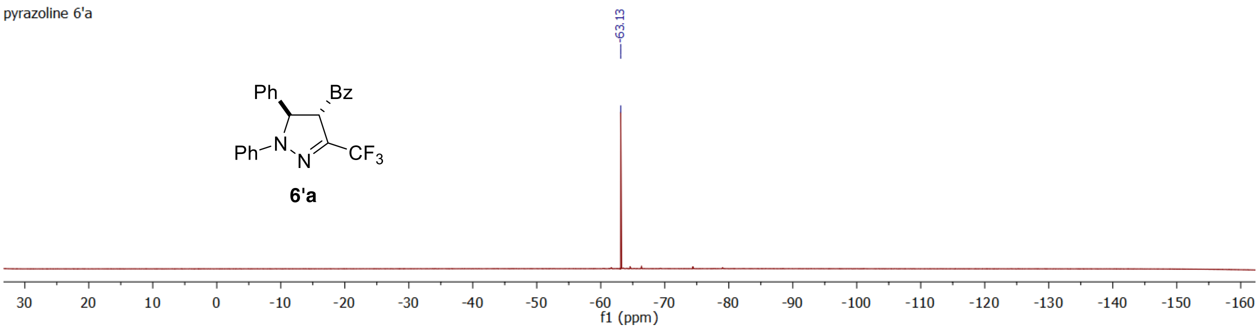


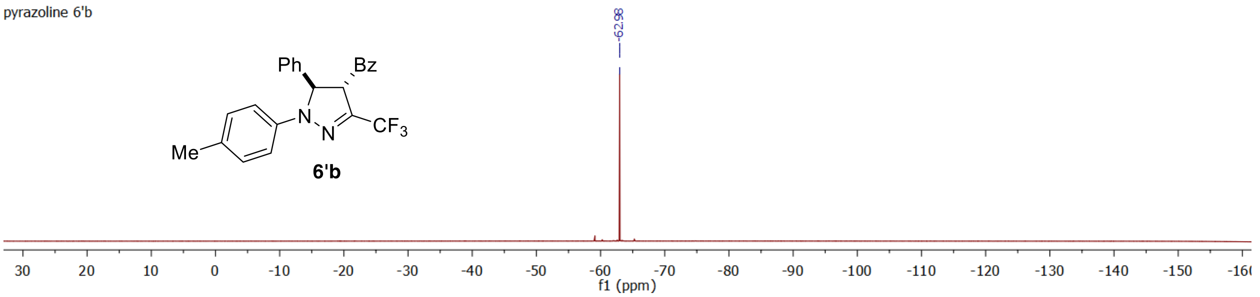


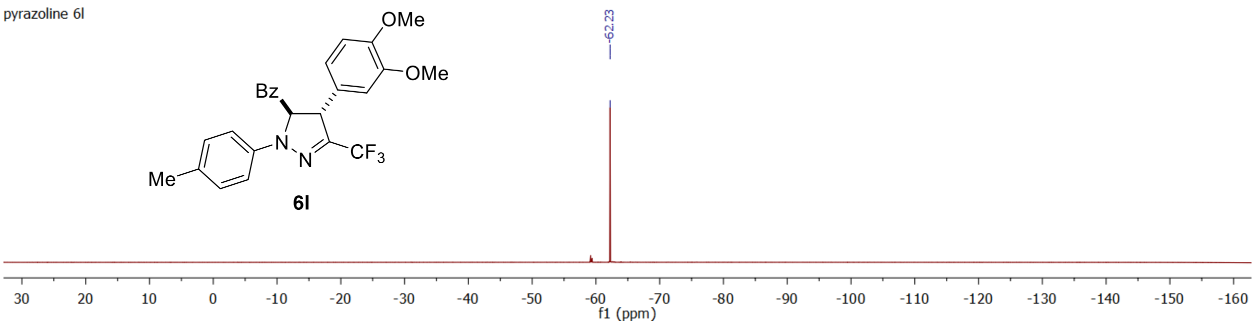


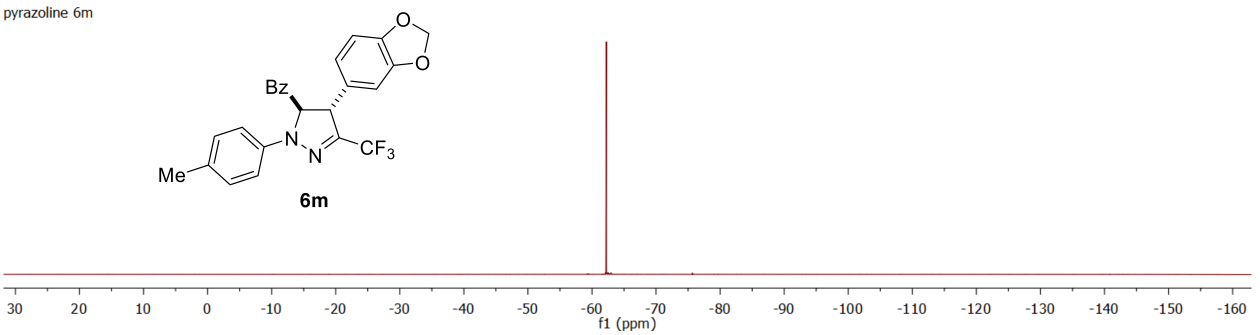


**Figure S21**. $^{19}$F NMR (565 MHz, $CDCl_3$) spectra for pyrazolines **6'a**, **6'b**, **6l** and **6m**.

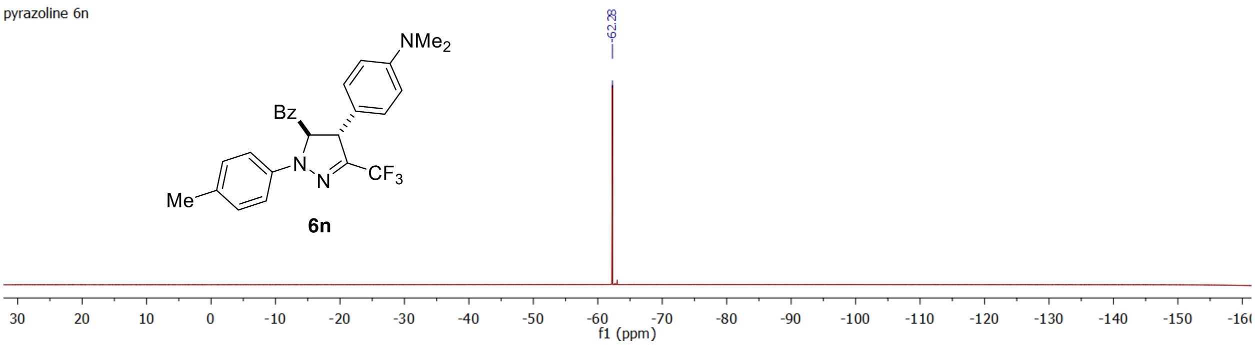


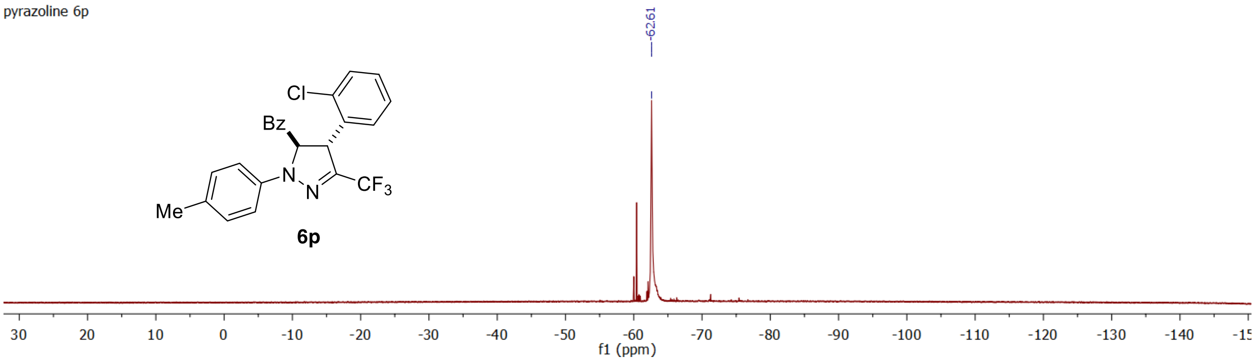


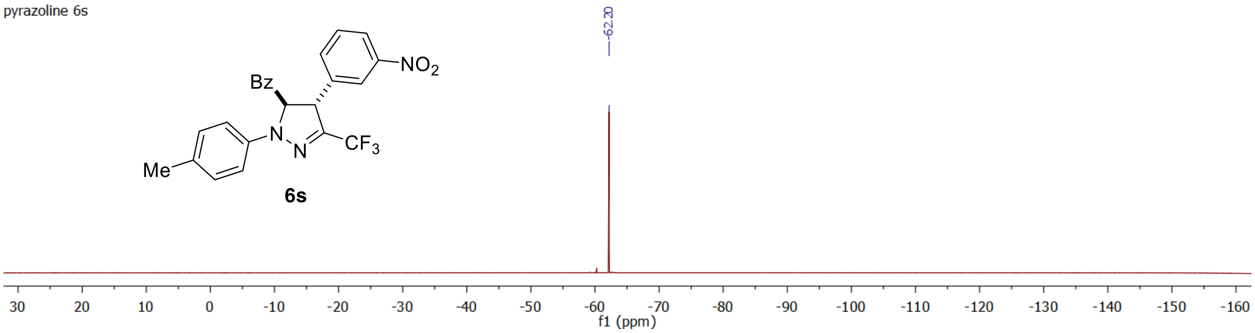


**Figure S22**. $^{19}$F NMR (565 MHz, $CDCl_3$) spectra for pyrazolines **6n**, **6p** and **6s**.

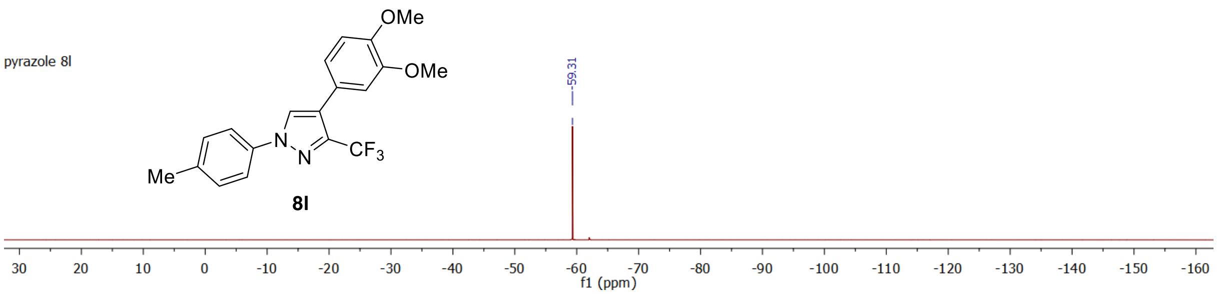


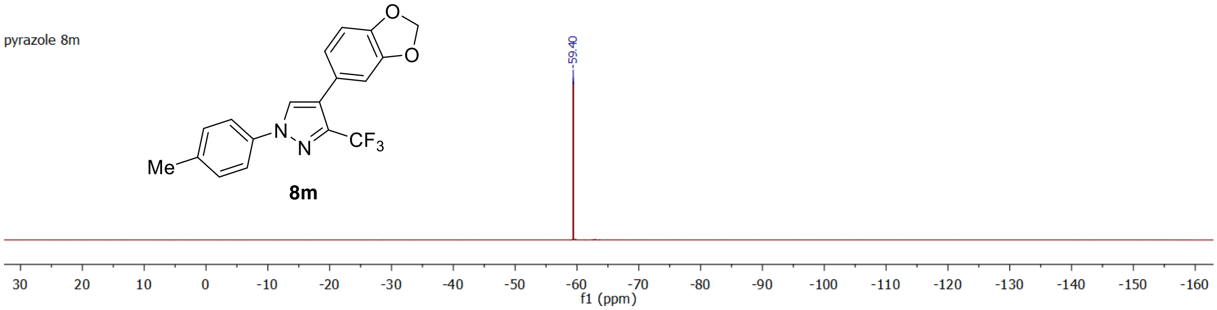


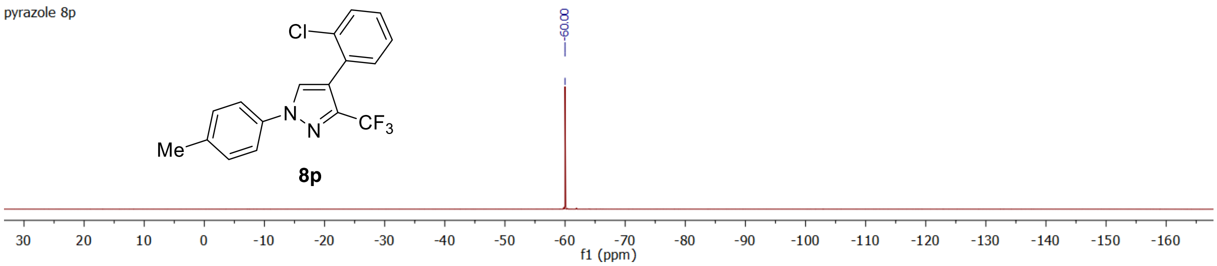


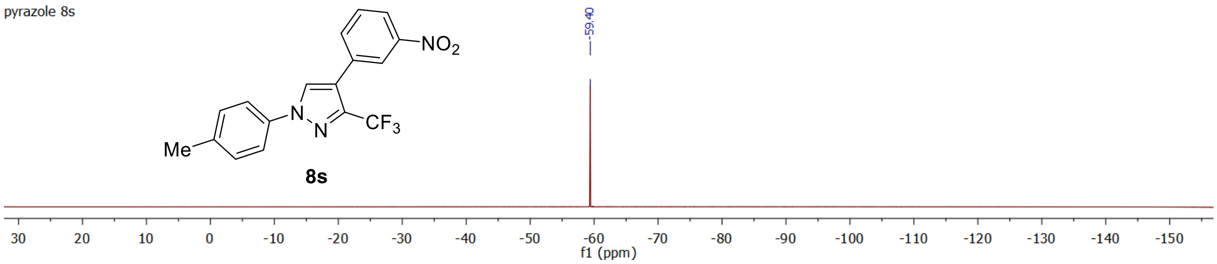


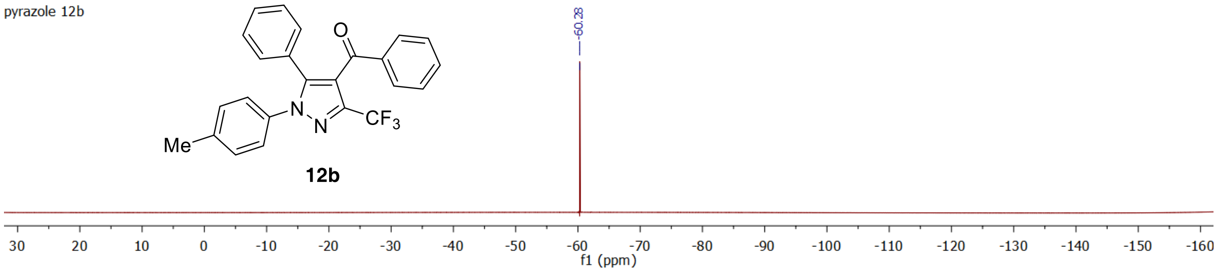


**Figure S23**. $^{19}$F NMR (565 MHz, $CDCl_3$) spectra for pyrazoles **8l**, **8m**, **8p**, **8s** and **12b**.
